# Episodes of Early Pleistocene West Antarctic Ice Sheet Retreat Recorded by Iceberg Alley Sediments

**DOI:** 10.1029/2022PA004433

**Published:** 2022-07-12

**Authors:** Ian Bailey, Sidney Hemming, Brendan T. Reilly, Gavyn Rollinson, Trevor Williams, Michael E. Weber, Maureen E. Raymo, Victoria L. Peck, Thomas A. Ronge, Stefanie Brachfeld, Suzanne O'Connell, Lisa Tauxe, Jonathan P. Warnock, Linda Armbrecht, Fabricio G. Cardillo, Zhiheng Du, Gerson Fauth, Marga Garcia, Anna Glueder, Michelle Guitard, Marcus Gutjahr, Iván Hernández‐Almeida, Frida S. Hoem, Ji‐Hwan Hwang, Mutsumi Iizuka, Yuji Kato, Bridget Kenlee, Yasmina M. Martos, Lara F. Pérez, Osamu Seki, Shubham Tripathi, Xufeng Zheng

**Affiliations:** ^1^ Camborne School of Mines University of Exeter Penryn Campus Cornwall UK; ^2^ Lamont‐Doherty Earth Observatory Columbia University Palisades NY USA; ^3^ Scripps Institution of Oceanography University of California San Diego La Jolla CA USA; ^4^ International Ocean Discovery Program Texas AM University College Station TX USA; ^5^ Department of Geochemistry and Petrology Institute for Geosciences University of Bonn Bonn Germany; ^6^ British Antarctic Survey Cambridge UK; ^7^ Alfred‐Wegener‐Institut Helmholtz‐Zentrum für Polar‐und Meeresforschung Bremerhaven Germany; ^8^ Earth and Environmental Studies Montclair State University Montclair NJ USA; ^9^ Department of Earth and Environmental Sciences Wesleyan University Middletown CT USA; ^10^ Department of Geoscience Indiana University of Pennsylvania Indiana PA USA; ^11^ Institute for Marine and Antarctic Studies University of Tasmania Battery Point TAS Australia; ^12^ Departmento Oceanografia Servicio de Hidrografia Naval Ministerio de Defensa Buenos Aires Argentina; ^13^ State Key Laboratory of Cryospheric Science Northwest Institute of Eco‐Environment and Resources Lanzhou China; ^14^ Geology Program University of Vale do Rio dos Sinos São Leopoldo Brazil; ^15^ Andalusian Institute of Earth Science (CSIC‐UGR) Granada Spain; ^16^ Cádiz Oceanographic Centre (IEO‐CSIC) Cádiz Spain; ^17^ College of Earth, Ocean, and Atmospheric Sciences Oregon State University Corvallis OR USA; ^18^ College of Marine Science University of South Florida St. Petersburg FL USA; ^19^ GEOMAR Helmholtz Centre for Ocean Research University of Kiel Kiel Germany; ^20^ Department of Earth Sciences ETH Zurich Zurich Switzerland; ^21^ Department of Earth Science, Marine Palynology and Paleoceanography Utrecht University Utrecht The Netherlands; ^22^ Earth Environmental Sciences Korea Basic Science Institute Chungbuk Cheongju Republic of Korea; ^23^ Knowledge Engineering Tokyo City University Tokyo Japan; ^24^ Faculty of Life and Environmental Sciences University of Tsukuba Tsukuba Japan; ^25^ Department of Earth Sciences University of California Riverside Riverside CA USA; ^26^ NASA Goddard Space Flight Center Planetary Magnetospheres Laboratory Greenbelt MD USA; ^27^ Department of Astronomy University of Maryland College Park MD USA; ^28^ Department of Marine Geology Geological Survey of Denmark and Greenland Aarhus University City Aarhus Denmark; ^29^ Institute of Low Temperature Science Hokkaido University Sapporo Japan; ^30^ Marine Stable Isotope Lab National Centre for Polar and Ocean Research Ministry of Earth Sciences Vasco Da Gama India; ^31^ South China Sea Institute of Oceanology Chinese Academy of Sciences Guangzhou China

**Keywords:** provenance, Ar‐Ar, SEM, QEMSCAN, MicroCT, IODP, scotia sea, pirie basin, southern ocean, ice rafted debris, Antarctic Ice Sheet, West Antarctic Ice Sheet, ice‐sheet retreat, deglaciation, IODP expedition 382, iceberg alley

## Abstract

Ice loss in the Southern Hemisphere has been greatest over the past 30 years in West Antarctica. The high sensitivity of this region to climate change has motivated geologists to examine marine sedimentary records for evidence of past episodes of West Antarctic Ice Sheet (WAIS) instability. Sediments accumulating in the Scotia Sea are useful to examine for this purpose because they receive iceberg‐rafted debris (IBRD) sourced from the Pacific‐ and Atlantic‐facing sectors of West Antarctica. Here we report on the sedimentology and provenance of the oldest of three cm‐scale coarse‐grained layers recovered from this sea at International Ocean Discovery Program Site U1538. These layers are preserved in opal‐rich sediments deposited ∼1.2 Ma during a relatively warm regional climate. Our microCT‐based analysis of the layer's in‐situ fabric confirms its ice‐rafted origin. We further infer that it is the product of an intense but short‐lived episode of IBRD deposition. Based on the petrography of its sand fraction and the Phanerozoic ^40^Ar/^39^Ar ages of hornblende and mica it contains, we conclude that the IBRD it contains was likely sourced from the Weddell Sea and/or Amundsen Sea embayment(s) of West Antarctica. We attribute the high concentrations of IBRD in these layers to “dirty” icebergs calved from the WAIS following its retreat inland from its modern grounding line. These layers also sit at the top of a ∼366‐m thick Pliocene and early Pleistocene sequence that is much more dropstone‐rich than its overlying sediments. We speculate this fact may reflect that WAIS mass‐balance was highly dynamic during the ∼41‐kyr (inter)glacial world.

## Key Points

- We present the first provenance data generated for Pleistocene‐aged iceberg‐rafted debris deposited in Iceberg Alley
- We conclude that prominent iceberg‐rafted debris layers deposited at Pirie Basin Site U1538 ∼1.2 Ma were sourced from West Antarctica
- They represent intense suborbitally‐paced episodes of iceberg discharge from tidewater glaciers, most likely in the Weddell Embayment

## 1 Introduction

The Antarctic ice sheets (AIS) are equivalent in volume today to ∼57.9 m of global sea‐level (Morlighem et al., 2020). The West Antarctic Ice Sheet (WAIS) contributes only ∼4.3 m to that total (Bamber et al., 2009). It is, nevertheless, considered to be more susceptible to climate change than the far larger East Antarctic Ice Sheet (EAIS). This is because the WAIS is largely (∼80%) marine‐based and ocean‐terminating (Bamber et al., 2018), which makes it unstable and prone to rapid retreat in response to atmosphere‐ocean warming (Schoof, 2007; Shepherd et al., 2019; Tauxe et al., 2015). Over the past 30 years, ice loss from the AIS was greatest, and accelerated fastest, in the WAIS (Shepherd et al., 2018). Such concerns have motivated some scientists to use ice‐sheet modeling to predict AIS response to future global warming scenarios (DeConto et al., 2021; Golledge et al., 2019). It has also motivated others to examine marine sediment cores from the Southern Ocean for evidence of AIS instability during the last deglaciation (e.g., Weber et al., 2014; Weber, Golledge, et al., 2021) and the warmer than present geological past (e.g., Bertram et al., 2018; Carlson et al., 2021; Carter et al., 2017; Cook et al., 2014, 2013; Gohl et al., 2021; Hansen et al., 2015; Jakob et al., 2020; Mckay et al., 2012; Naish et al., 2009; Passchier, 2011; Patterson et al., 2014; Scherer, 1991; Starr et al., 2021; Tauxe et al., 2015; Williams et al., 2010; Wilson et al., 2018).

**Figure 1:**
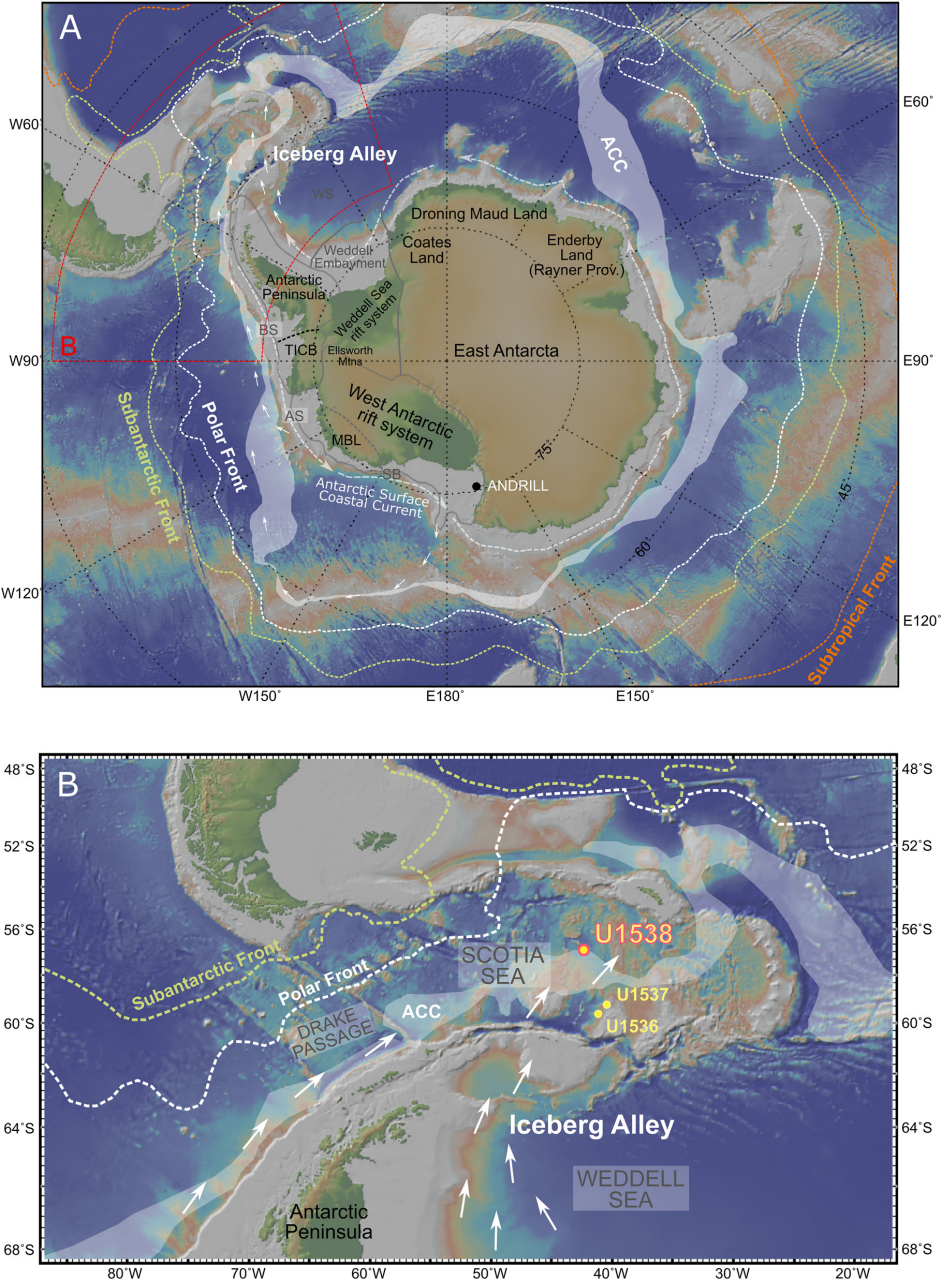
Study area: (a) South Pole projection of Antarctica and adjacent oceanography. Labels shown for main geotectonic provinces (gray lines), sub‐provinces (dashed lines), geographic regions mentioned in main text (BSE = Bellingshausen Sea Embayment; ASE = Amundsen Sea Embayment; SB = Sulzberger Bay; MBL = Marie Byrd Land) and key oceanographic fronts and currents (ACC = Antarctic Circumpolar Current); (b) Zoom‐in centered on Scotia Sea (red dashed box shown in panel a) showing location of the study site ‐ IODP Site U1538 (in Pirie Basin) and Dove Basin sites mentioned in the main text—IODP sites U1536/7 (Weber, Raymo, et al., 2021). Maps were created using GeoMapApp (http://www.geomapapp.org), the Global Multi‐Resolution Topography synthesis (Ryan et al., 2009) and oceanographic fronts and currents according to Orsi et al. (1995). White arrows in panels a and b show potential iceberg calving routes to Iceberg Alley according to Stuart and Long (2011) and Rackow et al. (2017).

The Scotia Sea is located in the Atlantic‐facing sector of the Southern Ocean and is ideally situated to preserve an integrated sedimentological record of AIS retreat. The Scotia Sea is otherwise known as “Iceberg Alley” (Anderson & Andrews, 1999) since it is the final destination for many AIS icebergs (Stuart & Long, 2011). Today, AIS icebergs converge within the Scotia Sea following their counterclockwise entrainment within the Antarctic Surface Coastal Current and the Weddell Gyre (Stuart & Long, 2011) and the clockwise flowing Antarctic Circumpolar Current (ACC) via Drake Passage (Rackow et al., 2017), where they melt in the relatively warm ACC surface waters shedding any iceberg‐rafted debris (IBRD) they contain to the sea floor (Figure 1). High‐resolution records of IBRD deposition in Iceberg Alley during the last deglacial have powerfully demonstrated the important role that AIS retreat played in centennial‐ to millennial‐scale phases of global sea‐level rise during Termination 1 (∼21–8 ka, e.g., Weber et al., 2014; Weber, Golledge, et al., 2021; Gomez et al., 2020). Nothing is known, however, about the Quaternary history of IBRD deposition in the Scotia Sea prior to this time. To date, provenance studies have also only been undertaken on IBRD deposited in Iceberg Alley during the Eocene (Carter et al., 2017).

In 2019, International Ocean Discovery Program (IODP) Expedition 382 drilled the most complete Plio‐Pleistocene sequences recovered to date from the Scotia Sea (Weber, Raymo, et al., 2021). IODP Site U1538 was drilled in Pirie Basin (Figure 1b), and its sequence represents one of the most expanded records of AIS‐proximal Pleistocene sedimentation ever recovered (Weber, Raymo, et al., 2021). Here we report the results of a preliminary investigation into the sedimentology and provenance of IBRD at Site U1538. We focus on the oldest of three early Pleistocene centimeter‐scale gravel‐ and sand‐rich layers recovered from this site. These layers are noteworthy for two reasons. First, they were identified by the shipboard scientists to be the most concentrated layers of IBRD in the Pleistocene sequence of Site U1538 (Weber, Raymo, et al., 2021). Second, the paleomagnetic stratigraphy for this site (Weber, Raymo, et al., 2021) indicates that these IBRD‐rich layers were deposited just prior to the Cobb‐Mountain subchron reversal (1.187–1.208 Ma; Channell et al., 2016) and therefore likely during the later part of marine isotopic stage (MIS) 38. This cold stage is notable as just one of two early Pleistocene cold stages for which convincing evidence has been presented for the existence of Last Glacial‐like Dansgaard‐Oeschger (DO) events during the ∼41‐kyr (inter)glacial world (Birner et al., 2016; Raymo et al., 1998). This evidence is found in marine paleoceanographic records from IODP Site U1385 in the Iberian margin, which for both the Last Glacial (e.g., Shackleton et al., 2000) and MIS 40 and MIS 38 (Birner et al., 2016) are shown to be characterized by sawtooth‐shaped ∼0.8–1.2‰ shifts in planktic foraminiferal (*Globigerina bulloides*) δ^18^O driven by millennial‐scale migrations in the Polar Front. Although other cold stages of the early Pleistocene are likely to have been characterized by DO‐like events (cf. Bailey et al., 2013; Gruetzner & Higgins, 2010; Hernández‐Almeida et al., 2012; Hodell & Channell, 2016), the Site U1538 record of MIS 38 provides a rare opportunity to examine potential northern and southern hemisphere linkages in suborbital changes in high‐latitude climate during a ∼41‐kyr (inter)glacial‐world cold stage proven to feature Last Glacial magnitude DO‐like events.

To this end, we present microCT‐imagery of the three‐dimensional fabric of our target layer which we use to confirm its iceberg‐rafted origin. We also characterize the petrography and mineralogy of its sand fraction using Scanning Electron Microscopy (SEM), and present the ^40^Ar/^39^Ar ages of sand‐sized hornblendes and biotite mica it contains to identify contributing AIS iceberg‐calving sources. We conclude that the IBRD in this layer was sourced from the West Antarctic Weddell and/or Amundsen Sea embayments and we discuss the glaciological significance of this finding.

## 2 Site U1538 Oceanographic Setting and Shipboard‐Derived Stratigraphy

Site U1538 was drilled in the northern part of Pirie Basin through a 3131‐m water column (at ∼57°S, 41°W; Weber, Raymo, et al., 2021). Sedimentation at this site is the product of pelagic aggregates (generated mainly by diatom productivity, and aeolian dust and IBRD deposition) and contour current deposition of mud‐sized material along the bottom‐current pathway of the ACC.

**Figure 2:**
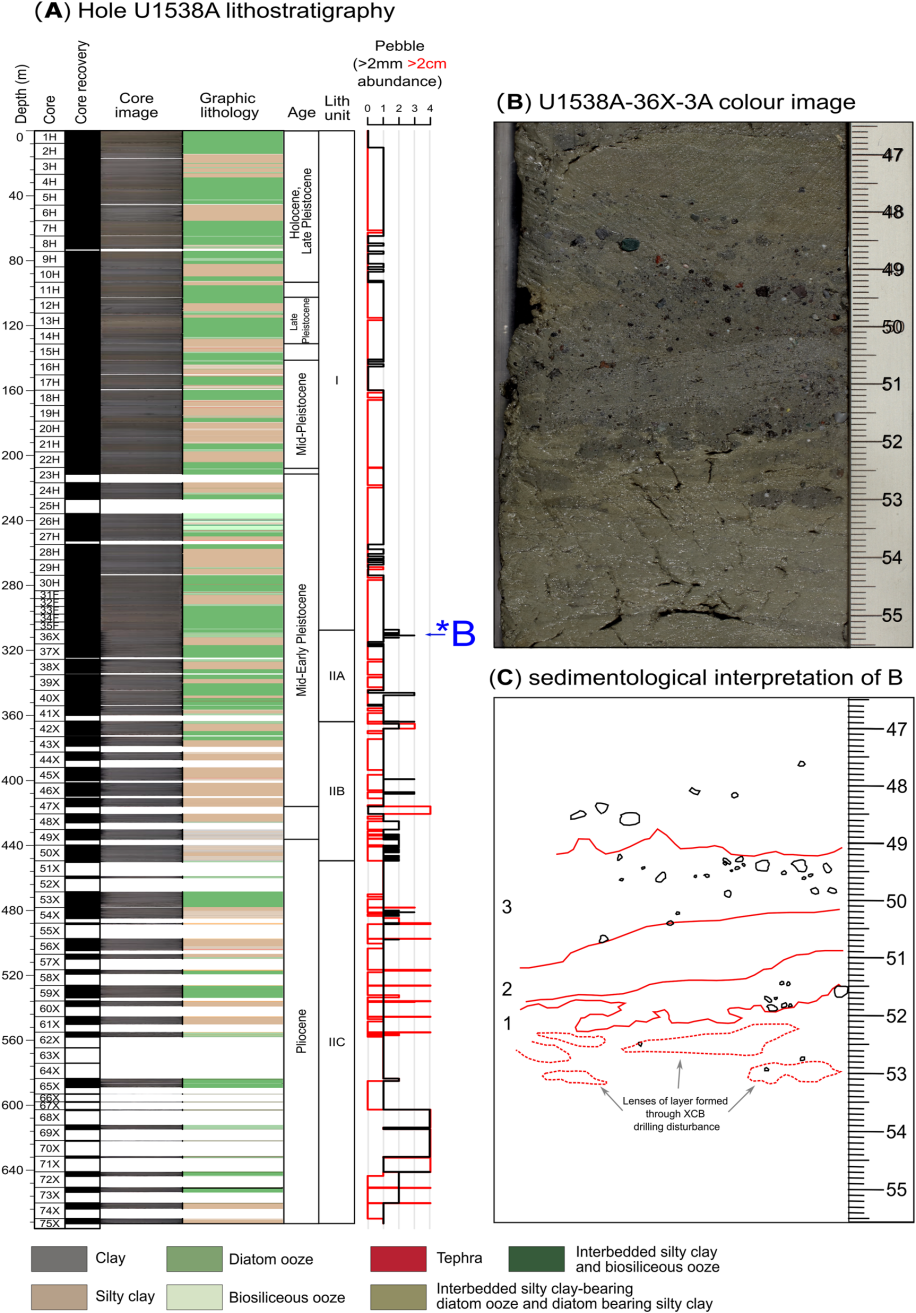
IODP Site U1538 Hole A lithostratigraphical summary: (a) lithostratigraphic units and shipboard‐derived ages, sedimentological descriptions and core photos, core recovery and iceberg‐rafted gravel (>2 mm) and pebble (>2 cm) abundance (Weber, Raymo, et al., 2021). Pebble abundance (>2‐mm/2‐cm) is semiquantitative visual assessment of concentration from 1 (no gravel or pebbles) to 5 (many gravel and pebble pieces) per section/core; (b) Color photo of the target gravel‐ and sand‐rich layer in Core 36X, Section 3 (scale bar is in cm; Weber, Raymo, et al., 2021); labeled as “*b” in panel (a); (c) Sedimentological interpretation of the layer shown in panel b Numbers 1–3 refer to position of three sublayers (or laminae) that make up the layer shown in panel b.

**Figure 3:**
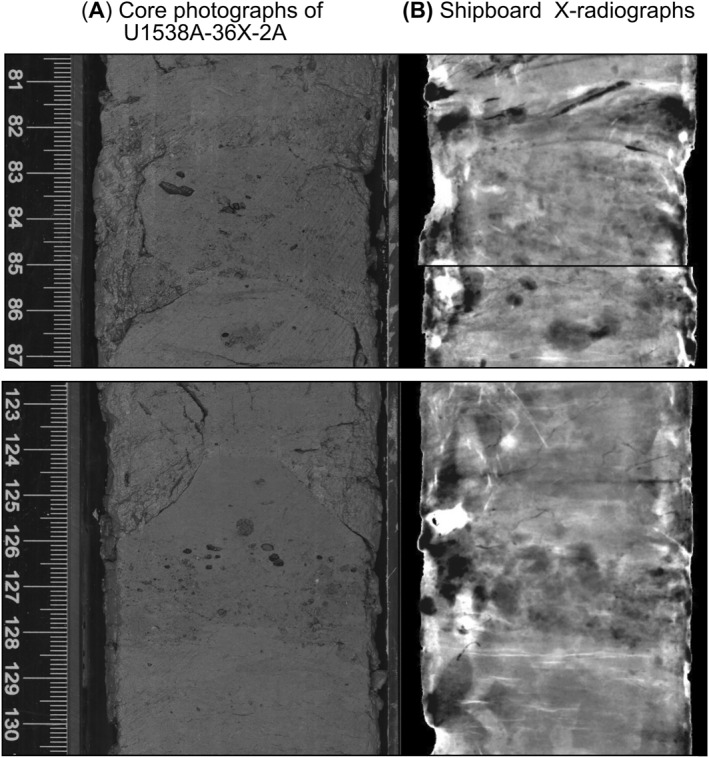
IBRD‐rich layers in U15385A‐36X‐2: (a) Core color photographs (Weber, Raymo, et al., 2021); (b) equivalent shipboard‐derived X‐ray images (Weber, Raymo, et al., 2021).

A spliced record for Site U1538 was constructed shipboard between 0 and 124 m composite depth (CSF‐D) from Holes U1538A, U1538C, and U1538D, but sediments were recovered down to 673 m below sea floor (mbsf) in Hole U1538A. The extended core barrel (XCB) system was required below 307.3 mbsf in Hole A. This depth broadly corresponds to the boundary between the shipboard‐defined lithostratigraphical units I and II, which marks the transition between lithified Pliocene and early to mid‐Pleistocene biosilicate‐bearing silty clays interbedded with silty clay‐bearing diatomites (Unit II) and mid‐ to late‐Pleistocene interbedded dark terrigenous‐bearing greenish‐gray diatom oozes and lithified biosilica‐bearing silty clays (Unit I; Figure 2a). Core descriptions highlight that dropstones and gravel‐ and sand‐sized IBRD are most abundant in Unit II (Figure 2a). Three discrete gravel‐ and sand‐rich layers 2‐to 4‐cm in thickness are preserved in sections 2 and 3 of Core 382‐U1538A‐36X (Figures 2 and 3). These layers have sharp lower and upper contacts and although they are preserved in diatom ooze, they are relatively diatom poor. The two youngest of these layers are found in Section 2 at 309.62–309.66 mbsf and 310.06–310.08 mbsf (Figure 3). The oldest and most prominent of these debris‐rich layers is found, however, at 310.79–310.82 mbsf in Section 3 (Figures 2b and 2c). All three layers were identified shipboard as being dominated by felsic (granitoid‐like) gravel‐sized clasts (Weber, Raymo, et al., 2021), but in this study we focus on examining the sedimentology and provenance of the best preserved of these ‐ the layer in Core 36X‐3 (Figures 2b and 2c). Incomplete core recovery below ∼354 mbsf (below Core 40X) prevents us from evaluating how common the deposition of these discrete IBRD layers was during the Pliocene and earliest Pleistocene at U1538, but shipboard descriptions demonstrate gravel and pebble concentrations may increase down hole toward its base (∼673 mbsf; Figure 2a). Unit II recovery is relatively low (ave. 69%, range 0%–110%), so some of these clasts may owe their origin to fall‐in down hole of late Pleistocene IBRD. We argue though, that the low recovery of Unit II sediments highlights how IBRD‐rich this unit is, and why the XCB system was required to facilitate its recovery. This suggestion is further substantiated by the fact that gravel and pebble concentrations reported shipboard for Dove Basin IODP sites U1536 and U1537 sediments are also highest in their Pliocene and early Pleistocene sections (Weber, Raymo, et al., 2021). Core recovery at these more southerly Scotia Sea sites was much higher than at U1538 and large portions of their Quaternary stratigraphies have been spliced (Weber, Raymo, et al., 2021, 2022). The higher concentrations of iceberg‐rafted gravels and pebbles in the Pliocene and early Pleistocene sequences of U1536 and U1537 therefore cannot be attributed to fall‐in.

**Figure 4:**
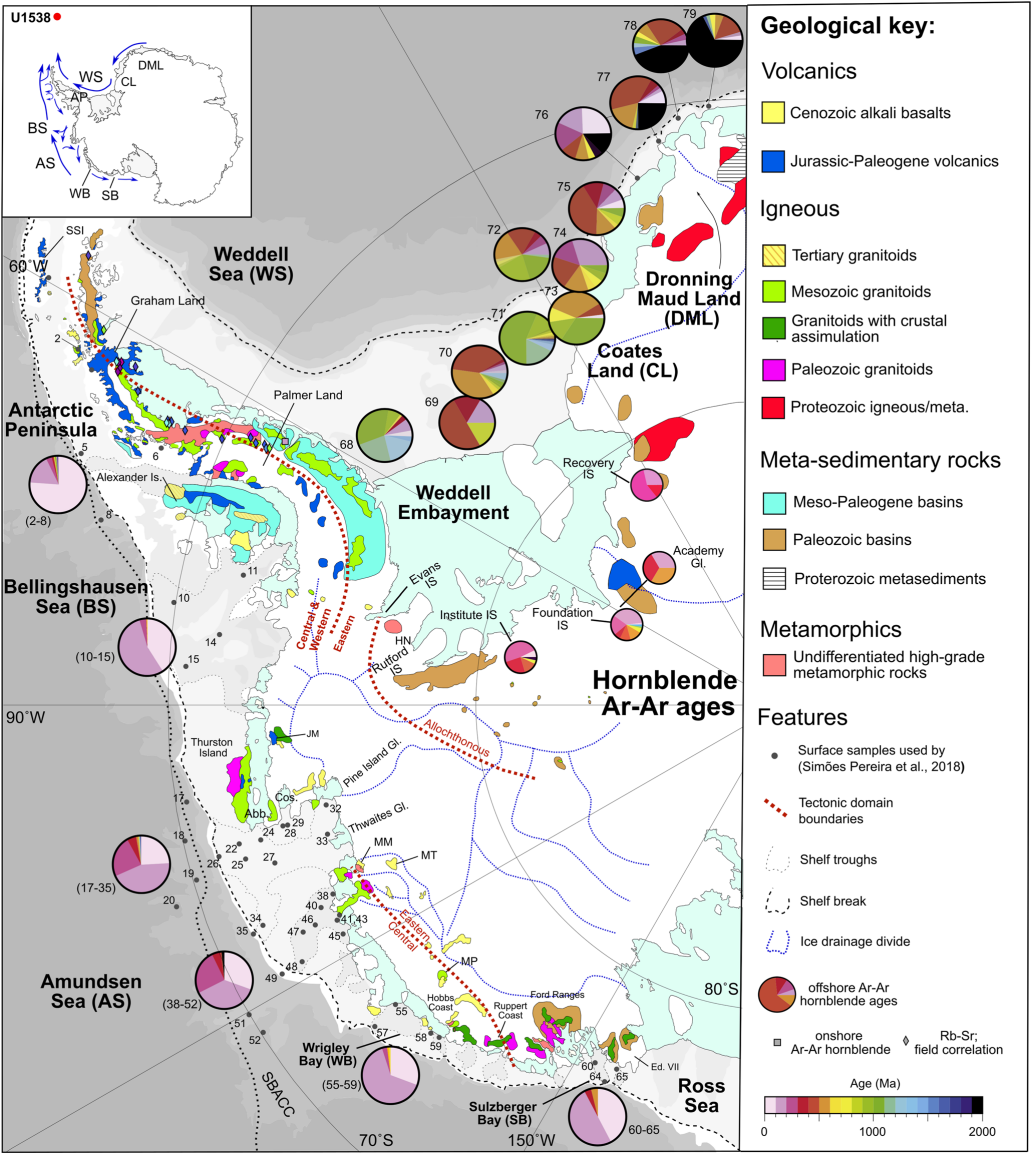
Simplified maps of West and East Antarctic geology and ^40^Ar/^39^Ar age provenance signature of sand‐sized hornblende grains from important modern‐day iceberg calving sources inferred in this study to supply the majority of IBRD deposited in Pirie Basin IODP Site U1538. Numbered small gray dots denote location of surface sediments analyzed by Simões Pereira et al. (2018)/Pierce et al. (2014) for Ar‐Ar ages summarized in largest‐sized pie charts shown. Ar‐Ar data summarized in smaller pie‐charts shown in the Weddell Embayment labeled Institute, Foundation, Academy and Recovery are based on analyses of hornblendes and biotites in till moraines (Agrios et al., 2021). Other onshore outcrop ages based on a compilation by Simões Pereira et al. (2018). Map insert shows main surface ocean currents (blue lines; Assmann et al., 2005; Gladstone et al., 2001; Murphy et al., 2013) directing icebergs to Site U1538. Also shown are major ice drainage divides (dashed blue lines), approximate boundaries between tectonic domains (dashed brown lines; Vaughan & Storey, 2000), bathymetric troughs (gray dashed line on the shelf breaks), the shelf break (dashed black lines), southern boundary of the Antarctic Circumpolar Current (and dotted line offshore; Orsi et al., 1995). Abbreviations: IS—Ice Stream; Abb—Abbot Ice Shelf; AS: Amundsen Sea; Cos—Cosgrove Ice Shelf; Dot—Dotson Ice Shelf; Ed. VII—Edward VII Peninsula: EWM—Ellsworth‐Whitmore Mountains; FR—Ford Ranges; JM—Jones Mts; HN—Haag Nunataks; HM—Hudson Mts; MM—Mt Murphy; MP—Mount Petras; MT—Mt Takahe; SSI—South Shetland Islands.; TI—Thurston Island; WC—Walgreen Coast. Map reproduced from Simões Pereira et al. (2018), with East Antarctic geology based on Pierce et al. (2014) and Bushnell (1971).

**Figure 5:**
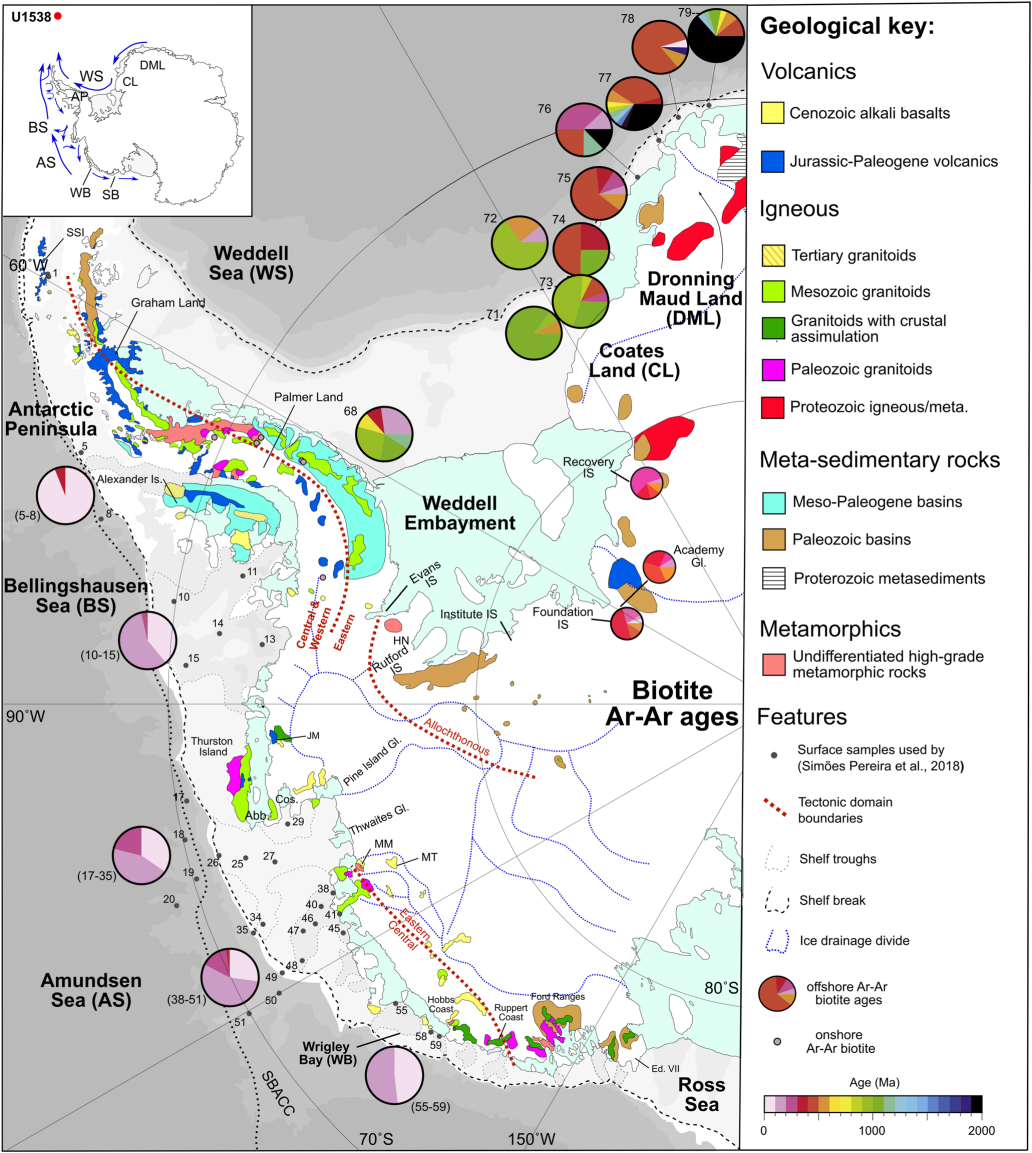
Simplified maps of West and East Antarctic geology and ^40^Ar/^39^Ar age provenance signature of sand‐sized biotite grains from important modern‐day iceberg calving sources inferred in this study to supply the majority of IRD deposited in Pirie Basin IODP Site U1538. Numbered small gray dots denote location of surface sediments analyzed by Simões Pereira et al. (2018)/Pierce et al. (2014) for Ar‐Ar ages summarized in largest‐sized pie charts shown. Ar‐Ar data summarized in smaller pie‐charts shown in the Weddell Embayment labeled Institute, Foundation Academy and Recovery are based on analyses of hornblendes and biotites in till moraines (Agrios et al., 2021). Other onshore outcrop ages based on a compilation by Simões Pereira et al. (2018). Map insert shows main surface ocean currents (blue lines; Assmann et al., 2005; Gladstone et al., 2001; Murphy et al., 2013) directing icebergs to Site U1538. Also shown are ‐ major ice drainage divides (dashed blue lines), approximate boundaries between tectonic domains (dashed brown lines; Vaughan & Storey, 2000), bathymetric troughs (gray dashed line on the shelf breaks), the shelf break (dashed black lines), southern boundary of the Antarctic Circumpolar Current (and dotted line offshore; Orsi et al., 1995). Abbreviations: IS ‐ Ice Stream; Abb—Abbot Ice Shelf; AS: Amundsen Sea; Cos—Cosgrove Ice Shelf; Dot—Dotson Ice Shelf; Ed. VII—Edward VII Peninsula: EWM—Ellsworth‐Whitmore Mountains; FR—Ford Ranges; JM—Jones Mts; HN—Haag Nunataks; HM—Hudson Mts; MM—Mt Murphy; MP—Mount Petras; MT—Mt Takahe; SSI—South Shetland Islands.; TI—Thurston Island; WC—Walgreen Coast. Map reproduced from Simões Pereira et al. (2018), with East Antarctic geology based on Pierce et al. (2014) and Bushnell (1971).

Although Site U1538 could theoretically receive IBRD from all major West and East Antarctic‐sources via anti‐clockwise transport of icebergs by the Antarctic Surface Coastal Current (Stuart & Long, 2011), iceberg monitoring (e.g., Budge & Long, 2018) highlights that four relatively proximal iceberg‐calving AIS sectors are likely to contribute the most to terrigenous deposition in Pirie Basin (Figures 1 and 4, 5). These are: (a) East Antarctic Dronning Maud Land and Coates Land, which are lined by relatively old Precambrian‐aged cratonic rocks (Pierce et al., 2014), (b) the adjacent Atlantic Ocean‐facing Weddell Sea Embayment that is occupied by ice streams that tap Cenozoic to Paleozoic geology underlying the EAIS and WAIS (Agrios et al., 2021), (c) the West Antarctic Peninsula that is dominantly composed of relatively young Cenozoic igneous bedrock (Simões Pereira et al., 2018; Jordan et al., 2020), and (d) a broad region of West Antarctica that includes the Amundsen Sea Embayment and forms the coastline between the Bellingshausen Sea and Sulzberger Bay (so is Pacific Ocean‐facing) that is composed of a complex and spatially diverse suite of Paleozoic meta‐sedimentary and meta‐volcanic rocks, Paleozoic, Mesozoic and Cenozoic plutonic igneous rocks and Jurassic to Cenozoic volcanics (Jordan et al., 2020; Simões Pereira et al., 2018). The pre‐Last Glacial Maximum (LGM) history of South Orkney Island glaciation is poorly constrained (see Dickens et al., 2014; Hodgson et al., 2014). This region of the sub‐Antarctic can probably be dismissed, however, as an important proximal contributor to IBRD deposition at U1538 during past glacials. This is because iceberg modeling studies highlight that icebergs in the Scotia Sea sourced from sub‐Antarctic landmasses are overwhelmed by iceberg outflow from the Weddell gyre through Iceberg Alley (Bigg, 2020). If any icebergs shed from the Pacific‐facing sector of the WAIS survive the journey to U1538, the largest tabular ones (so, size classes C4 and C5 of Wesche and Dierking (2015), ∼100–4700 km^2^, that are big enough to be tracked from space) mainly do so through anti‐clockwise transport in the Antarctic Surface Coastal Current (Stuart & Long, 2011). Many of the smaller ones (class sizes C1–3, 0.3–10 km^2^; Wesche & Dierking, 2015) may also do so through clockwise transport via the ACC through Drake Passage (Rackow et al., 2017). Importantly for this study, clockwise transport of abundant WAIS‐sourced icebergs to the Scotia Sea via the ACC is only viable for sites in the Pirie Basin (e.g., U1538) because this route is limited today for the other Scotia Sea sites drilled further south in, for example, Dove Basin during Exp. 382 (e.g., sites U1536/7; Figure 1b). According to iceberg trajectory modeling for the LGM (Starr et al., 2021), this route would likely have been absent for Dove Basin during Quaternary cold stages due to equatorward displacement of oceanic fronts in the ACC by northward‐shifted westerly winds (e.g., Kim et al., 2017).

**Figure 6:**
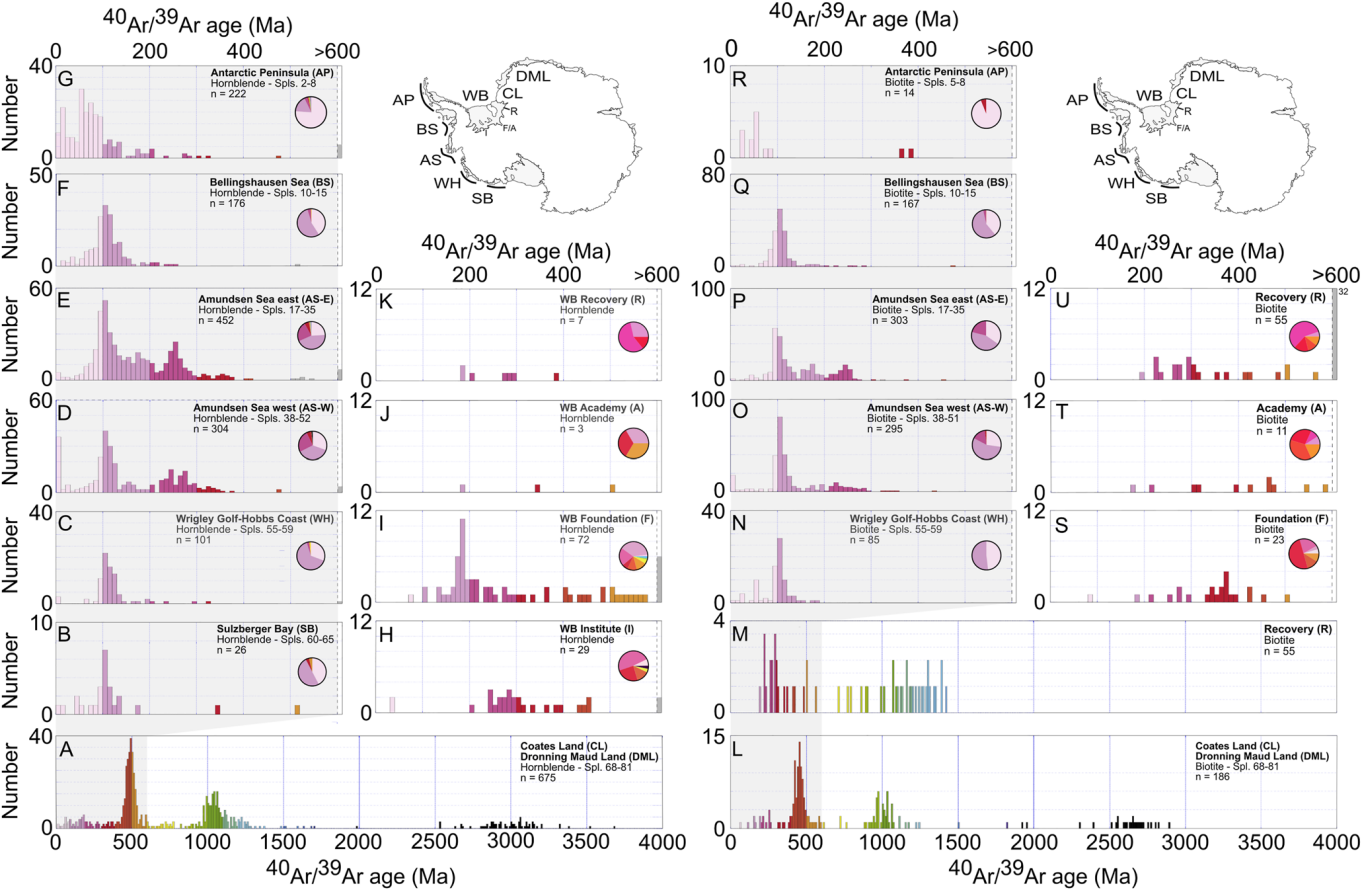
Provenance signature of modern Antarctic Ice Sheet iceberg‐rafted debris: histograms shown are of ^40^Ar/^39^Ar ages of sand‐sized iceberg‐rafted hornblendes (a–k) and biotites (l–u) from surface sediment samples adjacent to important modern‐day calving locations. Sample (Spls.) numbers in panels a–g and l–r refer to sample locations shown as gray circles in Figure 4. West Antarctica data for SB, WH, AS, BS and AP from Simões Pereira et al. (2018) and Roy et al. (2007). Weddell Bay (WB) data for F, A and R from Agrios et al. (2021). East Antarctic data for CL and DML from Pierce et al. (2014). Data in histograms binned every 10 Ma.

Only small areas of the Antarctic continent are not covered by ice. Thankfully, though, we can learn about the provenance signature of the bedrock underlying the drainage basin of glaciers feeding major modern‐day AIS iceberg‐calving sources indirectly by examining spatial variations in the age and composition of till moraines adjacent to key AIS ice streams (e.g., Agrios et al., 2021) and IBRD deposited in the AIS‐adjacent marine realm (e.g., Pierce et al., 2014; Perotti et al., 2017; Simões Pereira et al., 2018). The most comprehensive analyses to date of these ages are based on the ^40^Ar/^39^Ar ages of ice‐rafted hornblende, biotite and muscovite grains deposited adjacent to major EAIS (Agrios et al., 2021; Pierce et al., 2014) and WAIS (Simões Pereira et al., 2018; Agrios et al., 2021) iceberg sources (Figures 4, 5, 6).

## 3 Methods

### 3.1 Sampling, Sample Processing and Chronology

To determine the origin of the discrete IBRD layer in U1538A‐36X‐3W (from 310.79 to 310.82 mbsf) and the provenance of its coarse fraction, we sampled it using two orientated ∼13‐cm‐long u‐channels (2 × 2 × 13 cm). One of these u‐channels was used for microCT‐scanning to examine the layer's in‐situ sediment fabric, after which it was subsampled and washed over 63‐μm, 250‐μm and 1‐mm sieves to isolate different sand and gravel fractions of each of its three sublayers. To remove the biosilica from these coarse fractions, the samples were placed in LST (lithium heteropolytungstate) diluted to a density of 2.5 g/cc. The sinks from these samples were then subjected to LST density separation at 2.77 g/cc to aid picking of sand‐sized hornblende and mica grains for ^40^Ar/^39^Ar dating. The other u‐channel was subsampled at 1–2 cm resolution between 47 and 53 cm and all five resultant samples were washed over a 63‐μm sieve to isolate their sand fractions for SEM analysis of their mineralogy and sedimentology.

**Figure 7:**
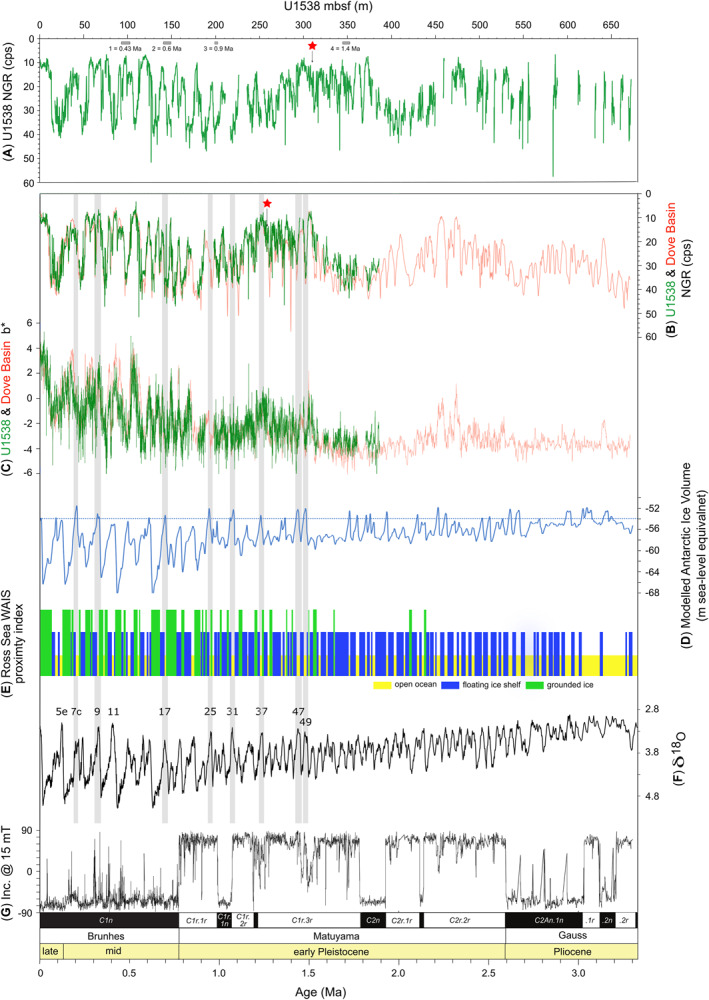
IODP Hole U1538A physical property records and other paleoclimate timeseries: (a) U1538A Natural Gamma Radiation (NGR) on coring depths (meters below seafloor = mbsf); (b) Same U1538A data as shown in a, but on ages following tuning (also see Data Set S5 in Bailey et al., 2022) of this site's NGR record to the Dove Basin (red data) NGR stack (Reilly et al., 2021); (c) U1538A sediment color‐b* data on the same age model as used for data in panel b; (d) Modeled Antarctic Ice volume (de Boer et al., 2015). Horizontal dashed blue line marks present day ice‐volume; (e) ANDRILL Ross Sea WAIS proximity index (Naish et al., 2009; yellow, open ocean; blue, floating ice shelf; green, grounded ice); (f) Global benthic δ^18^O stack (the LR04; Lisiecki & Raymo, 2005); (g) Raw shipboard‐derived archive‐half inclination data after 15 mT peak AF for IODP Site U1537 and interpretation of this magnetochron stratigraphy (Reilly et al., 2021). Red star in panel a/b highlights depth/age of the iceberg‐rafted debris layers reported in this study (also see Figure 8). Horizontal gray boxes in panel a labeled 1 to 4 show depth range uncertainty and published absolute ages for key Southern Ocean biozones identified shipboard in the U1538 stratigraphy (Weber, Raymo, et al., 2021): 1. Radiolarian Last Occurrence (LO) *Stylatractus universus* (93.27–102.56 mbsf), 2. Diatom LO *Actinocyclus ingens* (141.3–149.84 mbsf). 3. Diatom LO *Thalassiosira fasciculata* (198.55–202.98 mbsf). 4. Diatom First Occurrence *Fragilariopsis rhombica* (344.31–353.46 mbsf). Gaps in physical property data in panels a and b reflect core breaks in Hole U1538A. Numbers in panel e are marine isotope stage interglacials. Gray vertical bars highlight model‐based predictions of Antarctic ice‐volume reductions below present for the past 1.5 Ma shown in panel d. See Figure 1 for site locations.

To constrain the age of the IBRD layer we established tie lines between U1538 physical properties depth series and that of the magnetostratigraphically‐dated Dove Basin physical property stack (from nearby IODP sites U1536 and U1537) which preserves all magnetic reversals of the past 3.3 Myr with high fidelity in a nearly continuously spliced stratigraphy (Reilly et al., 2021). This was necessary because higher rates of sulfate reduction and hydrogen sulfide formation in U1538 sediments relative to other Scotia Sea Exp. 382 sites have led to dissolution of detrital magnetite and the in‐situ growth of the diagenetic magnetic mineral greigite, which complicates the interpretation of the magnetostratigraphy for this site (Weber, Raymo, et al., 2021). We chose to use shipboard‐derived natural gamma radiation (NGR) counts per second data to establish these ties because of the strong ∼1‐to‐1 temporal correspondence that is evident between NGR records from nearby sites U1356 and U1537 when placed on independent paleomagnetic‐based age models (Reilly et al., 2021; their figure 4). The remarkable similarity that exists between the Dove Basin NGR stack (Reilly et al., 2021) and the U1538 NGR depth‐series allowed us to establish 90 isochronous tie‐lines between these records (see Figure 7, and Data Set S5 in Bailey et al. (2022)).

NGR data were collected during Exp. 382 following standard IODP protocol (Blum, 1997; Weber, Raymo, et al., 2021) and have been used successfully to trace lithologic variability in Scotia Sea sediments (see Pérez et al., 2021; Reilly et al., 2021). NGR values are proportional to the sediment concentration of radioactive elements, particularly K, U, and Th (De Vleeschouwer et al., 2017). In settings like Dove and Pirie basins, the first order control on NGR is the relative contribution of biogenic (diatom) sediments (NGR = 0 counts per second (cps)) versus lithogenic sediments (NGR ≥0 cps). Opal‐rich sediments (or diatom oozes) are therefore associated with low NGR whereas silty‐clays are characterized by high NGR. Exp. 382 NGR records have a lower resolution (10 cm) than most other physical property data collected during the expedition (typically at 2 cm for spectral reflectance color data), but we used them to establish tie‐lines because they have a strong signal‐to‐noise ratio. The well‐documented linear relationship between the sediment color component b* and opal content of Scotia Sea sediments over the LGM‐Holocene (Sprenk et al., 2013) has recently been demonstrated to hold for sediments from this region over the past 1.4 Ma (Weber et al., 2022). We therefore also use shipboard color reflectance component b* records, as a first‐order proxy for opal‐rich sediments (with high b* values) and as an independent check on our NGR‐based tie‐lines.

### 3.2 X‐Ray Microtomography

X‐ray microtomography (microCT) is a non‐destructive 3D imaging and analysis technique for studying the internal structure of opaque samples (Wildenschild & Sheppard, 2013). Here we use microCT to characterize the 3D fabric of our IBRD‐rich layer. MicroCT data were collected on one of the ∼2 × 2 × 13 cm u‐channels of the layer at the Oregon State University microCT facility using a peak tube voltage of 120 keV and a 40 mA current. 3D images were generated using a helical scanning trajectory that allows for long scan sequences and fast acquisition time. Based on the sample geometry, a voxel (pixel) resolution of ∼14‐μm was achieved. The 7000+ projection images were reconstructed to produce a 3D volume of image intensities (where higher values indicate greater x‐ray attenuation), from which 2D cross‐sectional images can be viewed. Avizo software was used for 3D segmentation and volume rendering to visualize gravel and sand and create animations of the layer's 3D structure.

### 3.3 QEMSCAN® 4300 Scanning Electron Microscope Analyses

We determined the mineralogy of the sand fraction of the five samples extracted from the other u‐channel using a QEMSCAN® 4300 at the Camborne School of Mines, University of Exeter (Goodall & Scales, 2007; Gottlieb et al., 2000). QEMSCAN® permits estimates of the volume percentage of mineral grains present in a resin block of a disaggregated sample based on their chemistry determined using SEM energy‐dispersive X‐ray spectroscopy (EDS) analysis. Volume percentage estimates are generated for all mineral grains in the sample, whether present as isolated grains or as grains within lithic clasts of rock. The resultant output is used to determine what is known as “mineral association” and “lithotyping” data. Mineral association data provide a percentage estimate of mineral adjacency, so what other minerals each individual mineral identified touches (see Data Set S2 in Bailey et al. (2022)). Lithotyping data provide a percentage estimate of particle types (i.e., rock fragments composed of multiple grains).

To facilitate analyses, we mounted ∼1 g of the sand (>63‐μm) fraction of each sample in a 30‐mm diameter (15‐mm thick) epoxy resin (Epofix and Araldite) block. The face of each sample mount was then carefully ground and polished to expose the particles to a 1‐μm finish. Diamond‐based solutions were used in the polishing process to minimize contamination (because diamond is made of carbon it will not interfere with QEMSCAN® X‐ray analysis). Samples were then carbon coated using an Emitech K950 carbon coater to approximately 25‐nm to allow the electron beam to conduct across each sample surface.

Sample measurement and data processing were undertaken using the software packages iMeasure version 4.2SR1 and iDiscover 4.2SR1 and 4.3 (Rollinson et al., 2011). The QEMSCAN® settings used 25kV, 5nA, a 1000 X‐ray count rate per pixel, a working distance of ∼22‐mm under high vacuum and beam calibration every 30 min. We used the software's fieldscan measurement mode to analyze each sample (Pirrie & Rollinson, 2011) at an X‐ray resolution/pixel spacing of 8‐μm and a 1500‐μm^2^ field size (at x46 magnification).

Data processing and database development involved thoroughly checking every mineral grain category and adding and improving SIP (database) categories to match the composition of the samples analyzed. During this process, all mineral categories are checked (e.g., examination of elemental abundance, elemental ratios, backscattered electrons). The effects of excitation volume were also checked and boundary‐effect database entries added to handle these. The final part of data processing involved applying post processors. First, the fields were stitched together to create a mineral map of the entire sample area (about 27‐mm diameter; see Figures S1–S5 in Supporting Information S1). Second, a boundary phase processor was added to improve edge effects and remove rogue pixels. Third, a granulation processor was used to break particles out of the fixed image and then a touching particle processor was used to produce separate particles. This last step was required to permit the production of lithotyping data. The lithotyping data were generated using an image grid approach to filter the data digitally based on the particle characteristics of each sample. We used the particle visual and mineral data to help determine the categories used (see Data Set S3 in Bailey et al. (2022) for a description of the particle category criteria used). Once this was complete, the filters were copied to a chart to allow numerical data to be output along with the image grid. Data collection and processing followed in‐house quality control and quality assurance procedures. The data are estimated to be accurate to ∼50 ppm, below which confidence reduces due to the possibility of contamination.

### 3.4 ^40^Ar/^39^Ar Dating of Individual Iceberg‐Rafted Hornblende and Mica

All hornblende and mica (mostly biotite) were handpicked from the 250‐μm to 1‐mm size fraction, with additional mica picked from the 63–250‐μm size fraction. Hornblende, mica and standards were irradiated at the Cd‐lined in‐core facility (CLICIT) at the Oregon State reactor. ^40^Ar/^39^Ar ages were obtained using single‐step CO_2_ laser fusion at the Lamont Doherty Earth Observatory argon geochronology lab (AGES: Argon Geochronology for the Earth Sciences) to release argon, followed by cleanup with Zr‐Ar getters heated at 2‐amps. Extracted and cleaned gases were measured on a VG5400 noble gas mass spectrometer in peak hopping mode on an analogue multiplier, using the program Massspec. Nuclear interference corrections used values for OSU from Renne et al. (1998), and data were also corrected for background and mass discrimination using measured blanks and air pipettes. J values used to calculate ages were based on co‐irradiated Fish Canyon sanidine standard (28.201 ± 0.046 Ma; Kuiper et al. (2008), with decay constants from Min et al. (2000)).

## 4 Results

### 4.1 Stratigraphy

The results of our NGR tuning exercise between Hole U1538A and the Dove Basin stack are shown in Figure 7 (also see data set S5 in Bailey et al. (2022)). This exercise demonstrates that the top ∼425 m of Hole U1538A was deposited over the past ∼1.85 Ma (Figure 7b), corresponding to an average sedimentation rate of ∼23 cm ka^−1^. These facts are supported by the strong visual correlation that falls out independently from this tuning process between sediment color b* data from U1538 and the Dove Basin stack (Figure 7c). This exercise allows us to assign ages for all major chron reversals over this time from U1537 to the U1538A stratigraphy (Figure 7g).

**Figure 8:**
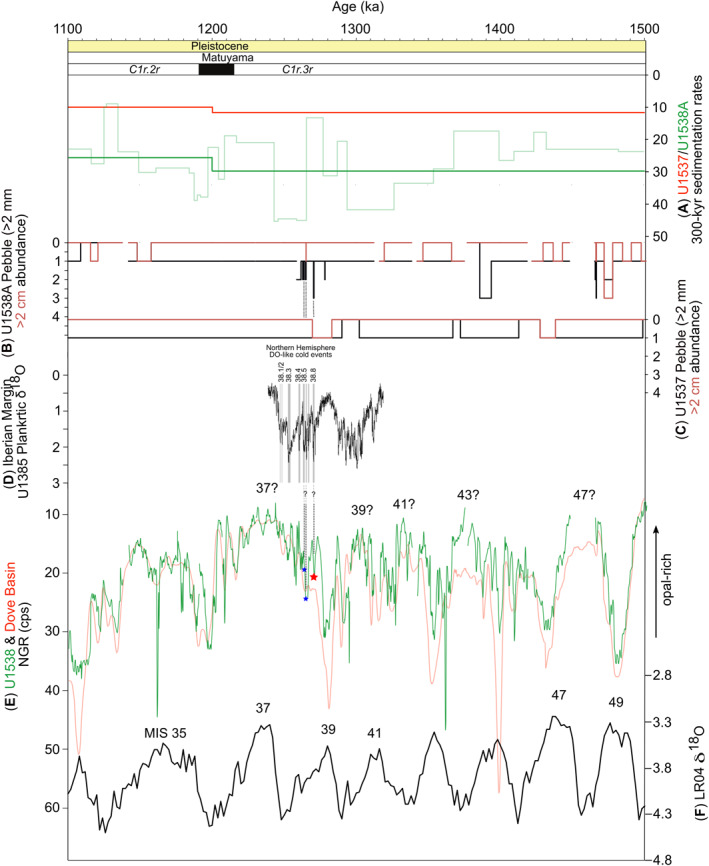
IODP Hole U1538A physical property records and other paleoclimate timeseries: (a) U1538A (bold green) and U1537 (bold red; Reilly et al., 2021) 300‐kyr averaged sedimentation rates (cm ka^−1^). Fainter green line is raw (i.e., not 300‐kyr averaged) U1538 sedimentation rates derived from U1538‐U1537 NGR ties (see Data Set S5 in Bailey et al. (2022)); (b and c) Shipboard‐inferred iceberg‐rafted gravel (>2 mm) and pebble (>2 cm) abundance (see Figure 2 caption for explanation; Weber, Raymo, et al., 2021); (d) Iberian Margin IODP Site U1385 *Globigerina bulloides* δ^18^O isotope record recording Last Glacial‐like Dansgaard‐Oescher (DO) events during MIS 40 and 38 (DO‐like northern hemisphere cold stadials are also highlighted for MIS 38 by numbered gray vertical bars; Birner et al., 2016); (e) U1538A (green) and Dove Basin stack (red; Reilly et al., 2021) NGR records; (f) Global benthic δ^18^O stack (the LR04; Lisiecki & Raymo, 2005). Also shown are locations of gravel‐ and sand‐rich layers in Core 36X, Section 2 (blue stars) and Section 3 (red star = focus of this study), and interpretation of magnetochron stratigraphy from IODP Site U1537 (Reilly et al., 2021).

Variations in U1538/Dove Basin NGR and LR04 benthic δ ^18^O data share many similarities over the past ∼1.5 Myr (compare Figures 7b and 7f). On the basis of their phase relations, it can be hypothesized that relatively opal‐rich sediments in Pirie and Dove basins (minima in NGR) correspond to warm stages (Reilly et al., 2021). While we refrain from performing additional tuning of the U1538 stratigraphy with interglacial‐glacial cycles in the LR04 stack, our new age model for this site allows us to identify that some diatom‐rich intervals (with relatively high/low b*‐color/NGR) were most likely deposited in the Scotia Sea during warm stages. Our age model suggests that diatom‐rich intervals (possibly indicative of a warm regional climate) likely correspond to all mid‐ to late‐Pleistocene interglacials of the past 500 ka (i.e., MIS 13, 11, 9, 7 and 5e; compare Figures 7b and 7f). Modeling of AIS extent suggests that early Pleistocene MIS 49, 47, 37, 31 and 25 may have been “super interglacials” and thus may have been characterized by substantial WAIS retreat relative to modern (Figure 7d; de Boer et al., 2015). Based on our age model, only one of these early Pleistocene interglacials (MIS 37) stands out, however, in our proxy‐opal records of NGR and b* as extra diatom‐rich intervals at U1538 (Figures 7b and 7c). We estimate that the three IBRD layers preserved in Core 36X were deposited ∼1.2 Ma and likely sometime during MIS 38 or the MIS 38/37 transition (Figure 8). It is hypothesized that the ∼41‐kyr pacing of early Pleistocene (inter)glacial cycles recorded by the LR04 stack owes its origin to the cancellation of precession signals by out‐of‐phase northern and southern hemisphere glaciations responding to the intensity of local summer insolation (the Antiphase Hypothesis; Raymo et al., 2006). Some data exist that suggest out‐of‐phase hemispheric precession signals were a feature of the 41‐kyr‐(inter)glacial world (e.g., Reilly et al., 2021; Scherer et al., 2008; Shakun et al., 2016). Although it is not a goal of this study to test this hypothesis, in light of its possible existence, we acknowledge that while our target layer may correspond to MIS 38 globally, NGR and color b* data from U1538 confirm shipboard sedimentological descriptions, and collectively they show that our target layer was deposited in opal‐rich sediments potentially indicative of a relatively warm regional climate.

### 4.2 Visual and MicroCT‐Imaging‐Based Description of Target Layer

**Figure 9:**
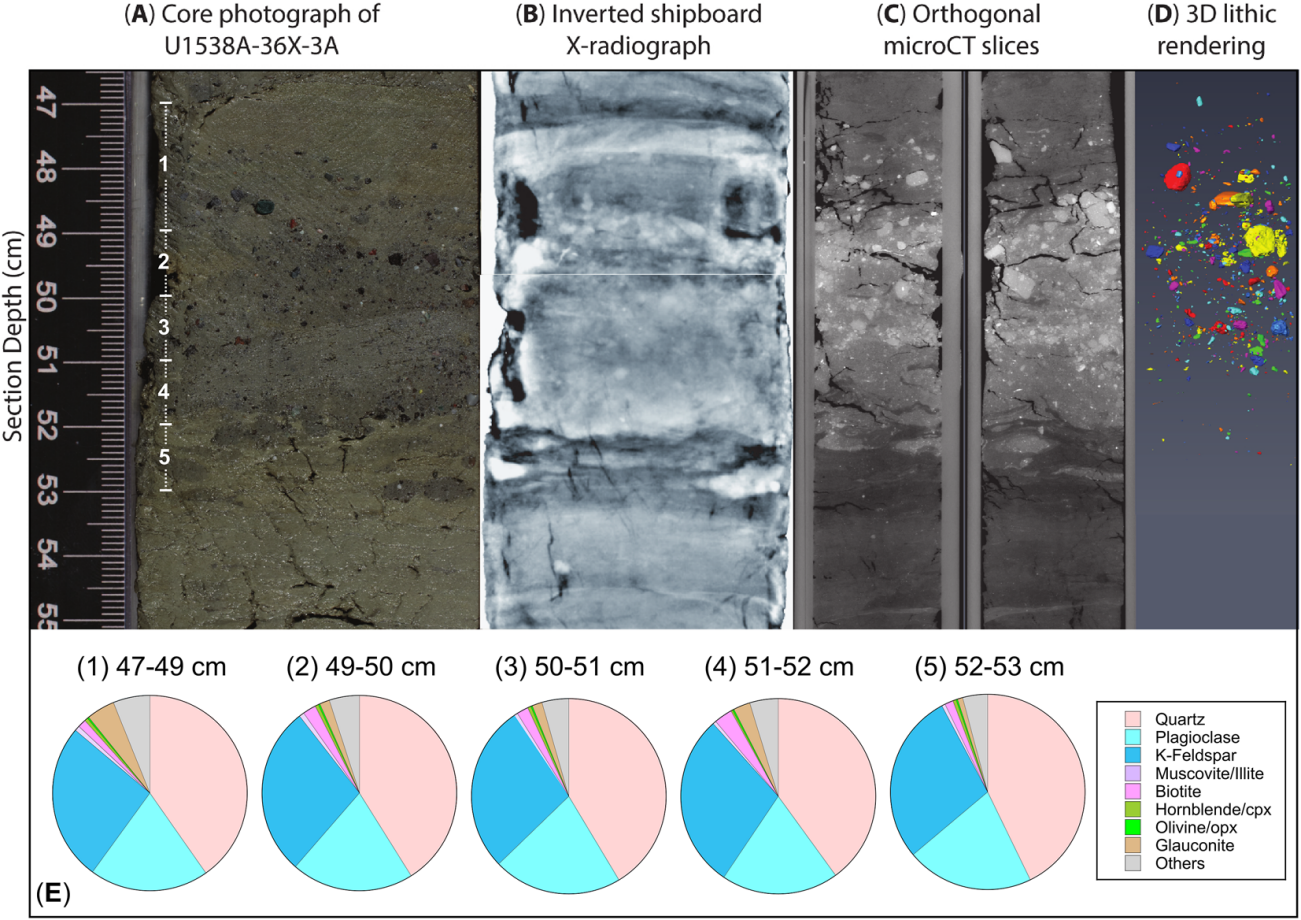
Images of IODP Hole A U1538A gravel‐ and sand‐rich layer in Core 36, Section 3: (a) Color photo of the layer (Weber, Raymo, et al., 2021); (b) equivalent color inverted X‐radiograph (Weber, Raymo, et al., 2021); (c) Orthogonal MicroCT scan image slices; (d) 3D rendering (from full suite of MicroCT imagery; also see Movie S1) of gravel‐sized clasts in the layer (color visualization of clasts is arbitrary); (e) QEMSCAN‐determined mineralogy of the sand fraction in the layer: Numbers 1–5 against pie charts in panel e correspond to sampling depths labeled in panel a.

Two orthogonal microCT‐based 2D cross‐sectional images and a 3D volume realization of our target IBRD layer are shown in Figure 9 alongside a high‐resolution color photo of it (also see Movie S1). This layer is ∼3‐cm thick, relatively diatom poor and has sub‐horizontal but moderately undulating sharp boundaries (Figure 9a). It is composed of three sublayers that are expressed as a dark‐light‐dark triplet. Sand‐sized terrigenous grains are present in all sublayers, but most of the gravel‐sized clasts are concentrated in the middle and uppermost sublayers (Figures 9c–9d). The undulating and sub‐horizontal nature of the layer's boundaries are most likely artifacts associated with XCB coring. We envisage a similar mechanism for the origin of the horizontal, millimeter‐scale elongated lenses that sit just below our target layer (between 52 and 53 cm, Figures 2b, 2c and 9a–9c). These are composed of the same sediment in the layer, but our microCT‐based 3D imagery rules out the only other plausible candidate for their origin ‐ bioturbation (see Movie S1). The preservation of a clear substructure within the layer itself suggests, however, that XCB coring has not destroyed its sedimentary fabric.

### 4.3 QEMSCAN®‐Based Quantification of Sand‐Fraction Mineralogy and Sedimentology

QEMSCAN®‐derived mineral compositions of the sand fraction of the IBRD layer are also presented in Figure 9e (see Data Set S1 in Bailey et al. (2022), and Table S1 in Supporting Information S1). The results demonstrate that >86% of the minerals identified in all samples are quartz (∼42%–44%), K‐feldspar (25%–28%) and plagioclase (20%–21%). A further ∼2%–3% are biotite and muscovite mica, ∼1%–5% glauconite and ∼0.5%–0.7% hornblende. Olivine and pyroxene are also present in all samples, but also only in low numbers (<1%). The samples are almost carbonate‐barren (<<1%). Accessory minerals common in granites and gneisses that are present include: apatite (0.15%–0.38%), garnets (0.4%–0.65%), zircon (0.01%), rutile (0.07%–0.1%), tourmaline (0.03%–0.06%), and Fe‐oxides (0.03%–0.38%); those common in basalts that are present include titanite (0.08%–0.24%) and ilmenite (0.05%–0.09%).

Our QEMSCAN®‐based mineral association analysis (see Data Set S2 in Bailey et al. (2022)), highlights that between ∼78%–83% of individual quartz, K‐feldspar and plagioclase identified in lithic clasts are in contact with each other. It also shows that at least 87% of the non‐isolated muscovite and 56% of non‐isolated biotite is in contact with either quartz, K‐feldspar or plagioclase. These mineral associations are reflected in the results of our lithotyping analysis (see data set S3 in Bailey et al. (2022)), which show that most of clasts identified are felsic in composition (between 88% and 95%), and are, for example, granitoids or clasts dominated either by quartz, K‐feldspar or plagioclase.

### 4.4 Ar‐Ar Ages of Ice‐Rafted Hornblende and Biotite Mica

**Figure 10:**
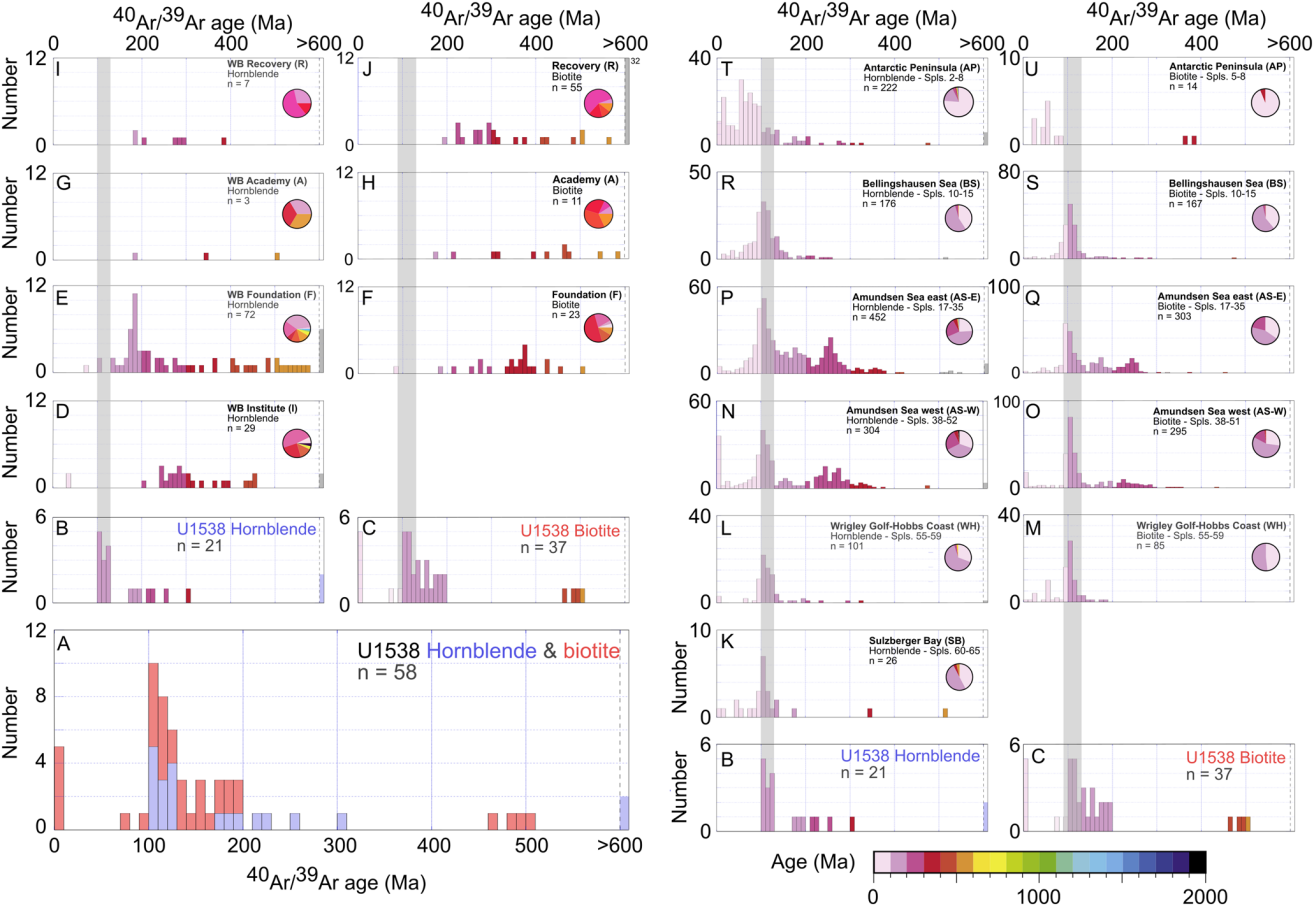
Comparison of ^40^Ar/^39^Ar ages of sand‐sized hornblende and biotite grains from IODP Hole U1538A gravel‐ and sand‐rich layer shown in Figures 2b and 2c & 9a and potential iceberg rafted sources: Ar‐Ar data from U1538A layer (this study; a–c). Ar‐Ar provenance signature of sand‐sized hornblendes (d, e, g, i) and biotite grains (f, h, j) from till moraines from important modern‐day Weddell Embayment iceberg calving sources (Agrios et al., 2021). Also shown is Ar‐Ar provenance signature of sand‐sized hornblendes (k, l, n, p, r, t) and biotite grains (m, o, q, s, u) from surface sediments deposited adjacent to important modern‐day iceberg calving sources on the Pacific‐facing sector of the West Antarctic Ice Sheet (Simões Pereira et al. (2018), Roy et al. (2007) and Pierce et al. (2014).

Histograms of the ^40^Ar/^39^Ar ages of 21 hornblende and 37 biotite grains from the middle sublayer are reported in Figure 10 (also see Data Set S4 in Bailey et al. (2022)). The majority of hornblende grains (12/21) have ages between 100 and 130 Ma with a second significant, but broader cluster of ages between 170 Ma and 310 Ma (7/21). Most biotite grains (33/37) are aged between 70 Ma and 200 Ma with a distinct peak centered on 100–120 Ma. The rest of the dated biotite grains are either younger than 10 Ma (5/37) or have ages between 460 and 510 Ma (4/37). The subtly different age profiles reported here for U1538 hornblende versus biotite grains likely reflect the different closure temperatures for argon of ∼550°C and ∼300°C, respectively (Harrison, 1982; Harrison et al., 1985), and the fact they are not found in all rock types in equal abundance. Most importantly, though, for our provenance inferences, all biotite grains and all but two of the hornblendes bear Phanerozoic ^40^Ar/^39^Ar ages. Just two hornblendes are older; one of these is Archean (3730 Ma) and the other is Proterozoic (∼810 Ma).

## 5 Discussion

### 5.1 Origin of the U1538 Early Pleistocene Gravel‐ and Sand‐Rich Layers

We can use the results of our sedimentological analysis of the oldest discrete U1538 gravel‐ and sand‐rich layer to evaluate the depositional processes responsible for its formation. These results highlight that the layer undoubtedly contains IBRD. The specific question we therefore need to address is whether this layer is the direct product of an intense interval of AIS‐iceberg discharge and IBRD deposition in the Pirie Basin (as inferred shipboard) or whether it represents reworking of IBRD by a gravity flow, or the concentration of the gravel‐ and sand‐sized IBRD by high‐velocity bottom‐current winnowing of the mud fraction (i.e., of clay and sortable‐silt).

At a first glance, the IBRD layer's sedimentology appears broadly similar to that ascribed to the sedimentary products of a debris flow –that is, a debrite (a poorly sorted bed containing gravel and sand in a mud‐rich matrix; e.g., Cukur et al., 2021). Yet the layer's internal structure (the dark‐light‐dark triplet of sublayers) is inconsistent with having been deposited as a single unit/in a one‐off event. Similarly, the layer superficially resembles the S1 or S2 divisions of the *Lowe* sequence of coarse‐grained turbidites, which can be characterized by structureless (ungraded) sandy gravels/pebbly sands (Lowe, 1982). But unlike our target layer, these divisions of the *Lowe* sequence should be devoid of mud (Lowe, 1982). Gravel‐sized clasts in turbidites often also exhibit long‐axis alignment and up‐flow inclined imbrication (Stow & Smillie, 2020), yet no such fabric is evident in our 3D realization of the U1538 layer (Figure 9d and Movie S1). The layer we have studied is also only ∼3 cm thick whereas gravel‐rich turbidites, which are deposited in confined submarine channel flows in the inner fan, tend to be of at least an order of magnitude thicker (Lowe, 1982).

**Figure 11:**
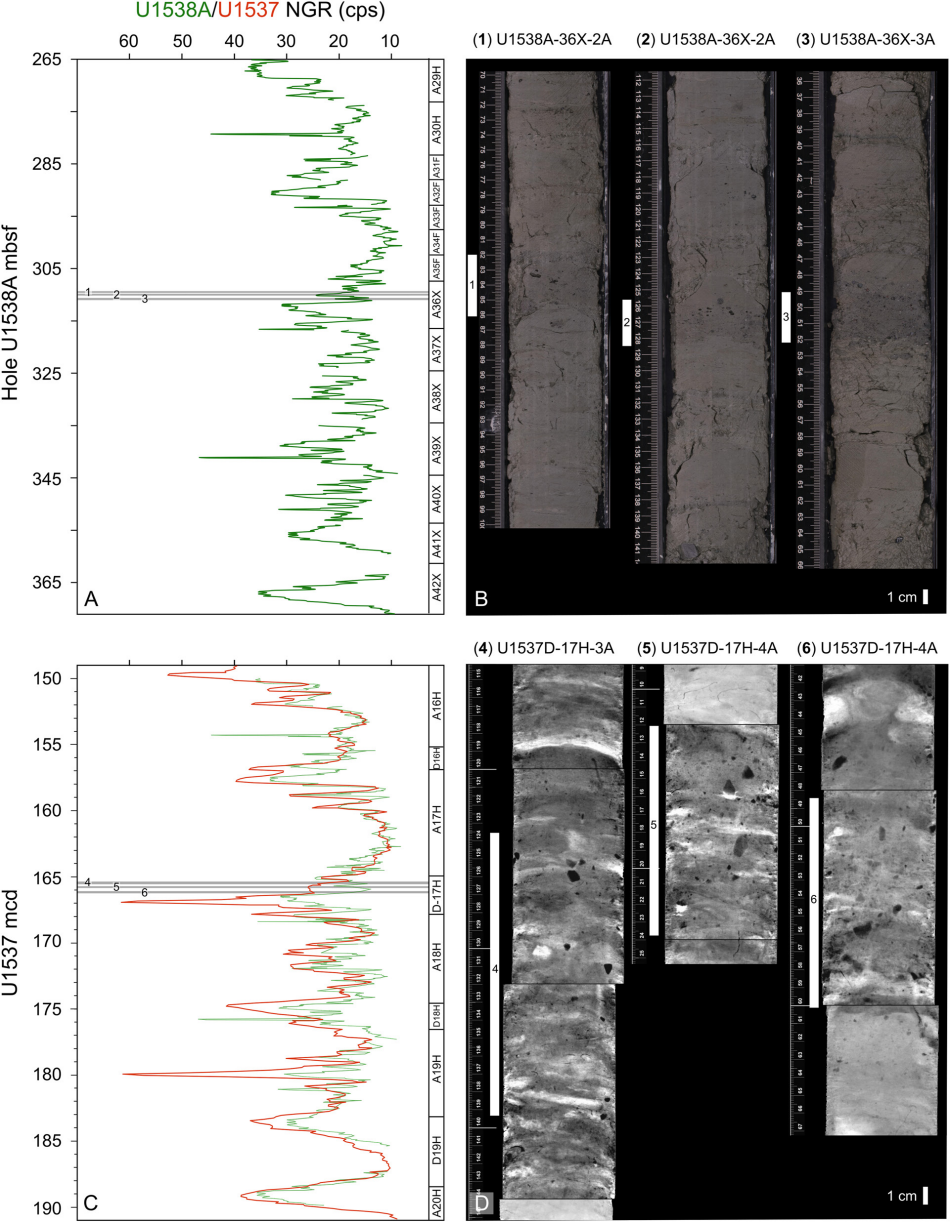
Temporal relationship between early Pleistocene Scotia Sea IBRD‐rich intervals deposited at two Scotia Sea sites during the early Pleistocene: Core color photos of three discrete IBRD‐rich layers reported in this study from Pirie Basin Site U1538A (b; vertical white bars labeled 1–3) and X‐ray images of what we infer to be broadly time‐isochronous horizons with abundant diffuse IBRD (black mm‐ to cm‐scale grains in X‐ray images) from Dove Basin Site U1537 (d; vertical white bars labeled 4–6). This correlation is based on comparison of Natural Gamma Ray (NGR) count per second (cps) data shown in (a and c) (also see Figure 7 and Section 3.1). Note—U1538 NGR data on mbsf in panel a are also shown in panel c on U1537 mcd assigned to these data using NGR ties listed in Data Set S5 in Bailey et al. (2022). Also note—unlike at U1537, the IBRD‐rich layers from U1538A are not clear in their corresponding core X‐ray images because the sequence at this depth (∼310 mbsf) is composed of semi‐indurated sediments. mbsf = meters below seafloor; mcd = meters composite depth. All data and images shown from Weber Raymo, et al. (2021).

Sedimentation at Site U1538 is dominated by pelagic aggregates and lateral advection of mud‐sized sediment by contour currents. If these bottom currents are sufficiently vigorous, diatom‐ooze‐rich sediments containing low concentrations of IBRD can be stripped of mud (the fraction in which most diatom frustules reside; Round et al., 1990) to leave behind a condensed sequence of concentrated sand and gravel. We find this scenario is unlikely, however, to explain the origin of our IBRD layer because it has a high mud content (of ∼20%–40%). Bottom current speeds at U1538 were higher during the mid‐ to late‐Pleistocene than during the early Pleistocene (Pérez et al., 2021). If bottom currents at U1538 were regularly strong enough to cause pronounced mud winnowing we might therefore expect to find many examples of these layers in late Pleistocene sediments deposited at this site for which recovery is high, but we do not (Figure 2a). Moreover, if high velocity mud winnowing was a common feature of the U1538 depositional environment, its stratigraphy would feature multiple hiatuses and/or condensed horizons, which would be incompatible with the strong regional coherence that exists in physical property cycles between Scotia Sea records (Pérez et al., 2021; Reilly et al., 2021; Weber, Raymo, et al., 2021). We therefore conclude that our IBRD layer is most likely the sedimentary product of an intense but short‐lived episode of AIS‐iceberg rafting and IBRD deposition. This inference is supported by shipboard‐derived X‐ray images of the Site U1537 sequence, which reveal three sediment horizons (labeled 4–6 in Figures 11c and 11d) with abundant but disseminated gravel that, based on our U1537‐U1538 NGR‐based correlations (Figures 11a and 11c), were deposited in Dove Basin at the same time as the three early Pleistocene IBRD layers from Site U1538 (labeled 1–3 in Figures 11a and 11b). This is because events of this size would be unlikely to deposit IBRD at just one site.

### 5.2 Provenance of Pirie Basin Iceberg‐Rafted Debris‐Rich Layer

To our knowledge, the three IBRD layers preserved in Hole U1538A‐Core 36X are the first discrete Quaternary‐aged IBRD layers discovered in warm‐stage (i.e., opal‐rich) Iceberg Alley sediments. We can determine the provenance of the IBRD in the oldest of these layers by comparing the ^40^Ar/^39^Ar ages of hornblende and biotite grains it contains to those deposited near major modern‐day AIS iceberg‐calving sources (Figure 10).

The strong dominance of Phanerozoic grains in our target layer (*n* = 56/58) strongly implies that the vast majority of IBRD in it was derived from West Antarctica (Figure 10). Ice‐sheet modeling for the AIS during the early Pleistocene highlights that the Antarctic Peninsula was glaciated during early Pleistocene cold stages (Pollard & DeConto, 2009). Yet the absence of large numbers of early/middle Cenozoic grains our U1538 IBRD may rule out this region of Antarctica from being the primary source involved (compare Figures 10b to 10t and 10c to 10u). No ^40^Ar/^39^Ar ages exist for IBRD in surface sediments deposited off the Atlantic‐facing sector of the Antarctic Peninsula. Terrestrial sequences from this region (the Eastern Domain in Figure 4) feature ages spanning ∼170–250 Ma based on a variety of dating methods (see small colored diamonds in Figure 4). Until ^40^Ar/^39^Ar grain ages are acquired for surface sediments from this region (e.g., north of sample 68 in Figure 4) we cannot therefore exclude the “Eastern Domain” of the Antarctic Peninsula as a source of our IBRD. Based on the available source provenance data, we can nevertheless be confident that the EAIS was not the primary source of the IBRD in our target layer. Indeed, when compared to the available source data, the distinct modal peak of late Cretaceous ages (of 100–130 Ma) in the hornblende and biotite populations from our target layer with a positively skewed tail of Carboniferous to Jurassic ages (∼130–310 Ma) most strongly matches the provenance signature of the Amundsen Sea and/or Weddell Sea embayments (see Figure 10). Ice‐rafted hornblendes and biotite grains in Amundsen Sea Embayment core‐top sediments are easily distinguished from those derived from other major WAIS iceberg caving sources on its Pacific‐facing side because many of them possess ages >140 Ma (Simões Pereira et al., 2018). The geology underlying Pine Island and Thwaites glaciers adjacent to Amundsen Sea Embayment is dominated by Mesozoic (grano)diorites and "pink’ granites (White & Craddock, 1987). This suite of rocks matches up well with the mineralogy of the detrital sand‐fraction of our U1538 layer. All the sand we analyzed from this layer is dominated by quartz, K‐feldspar and plagioclase and contain accessory minerals common in granites and gneisses (e.g., apatite, garnet, zircon, rutile and tourmaline) ‐ either as isolated grains or as part of granitoid‐ and/or gneiss‐like lithic clasts. The provenance signature of WAIS iceberg‐calving sources in the Weddell Embayment is less well resolved but could also feasibly be responsible for the age distribution of the hornblende and mica found in our target layer (Figure 10). Carboniferous to Jurassic (but not late Cretaceous) aged grains appear to characterize bedrock eroded by the Institute Ice Stream (Figure 10d). IBRD source data are currently lacking for the westernmost (Rutford and Evans) ice streams draining the WAIS on Palmer Land (Agrios et al., 2021). Limited onshore bedrock hornblende and biotite ages for this region of West Antarctica do, however, yield ∼100 Ma ages (see small‐sized onshore squares (hornblende) and circles (biotite) in Figures 4 and 5; Simões Pereira et al., 2018).

The absence of discrete IBRD layers at corresponding stratigraphic depths at the more southerly Dove Basin Iceberg Alley sites (at U1536/7; Weber, Raymo, et al., 2021) may highlight that WAIS‐sourced iceberg‐rafting via Drake Passage best explains our provenance data. Alternatively, the higher IBRD concentrations at U1538 may simply reflect that most northward‐drifting icebergs in Iceberg Alley during the early Pleistocene melted in Pirie Basin and not the more southerly Dove Basin. It is also likely that many IBRD in icebergs calved into the Amundsen Sea Embayment would melt out during any counterclockwise journey around Antarctica in the Antarctic Surface Coastal Current before reaching Iceberg Alley. Even if many of them survived the journey, the provenance signature of any IBRD they contained would likely be diluted significantly by IBRD calved from EAIS and WAIS sectors more proximal to the Scotia Sea (as observed in provenance studies of Greenland‐sourced IBRD; e.g., White et al., 2016). A more‐direct clockwise transit through Drake Passage in the ACC is more likely to result in large numbers of Amundsen Sea Embayment icebergs dominating U1538 IBRD deposition (Rackow et al., 2017; their Figure 2). The two sand‐sized hornblendes we report from our U1538 layer bearing Archean (∼3730 Ma) and Proterozoic (∼810 Ma) ages and thus could conceivably be derived from East Antarctic Maud Land or Raynor provinces (Pierce et al., 2014) or the Filchner Ice Stream of the eastern Weddell Embayment (Agrios et al., 2021). We note, though, that small numbers of Proterozoic hornblendes (including an age cluster of ∼640–800 Ma) are also reported from Amundsen Sea core‐top sediments (Simões Pereira et al., 2018). Proterozoic grains could also be sourced from the Rutford and Evans ice streams in the western Weddell Embayment. This is because magnetic anomaly data from this region are interpreted to show that the Proterozoic Haag basement, which extends beneath the Ellsworth Mountains, lies beneath these two ice streams (Maslany & Storey, 1990). On its eastern side, the Rutford Ice Stream also flows adjacent to a Paleozoic sedimentary basin in the Ellsworth Mountains that could contain reworked Proterozoic grains (Figure 4).

### 5.3 Glaciological and Climatological Consequences of the Pirie Basin Ice‐Rafted Debris Layers

Our discovery of evidence for early Pleistocene episodes of intense iceberg discharge from the WAIS to Iceberg Alley raises questions about their glaciological and climatological significance. The three discrete IBRD layers we report from Core 36X are relatively thin (just 2–4 cm thick), relatively diatom poor, and are preserved in an opal‐rich interval which we infer was deposited during a regional warm‐stage climate ∼1.2 Ma. This sedimentological context points toward their deposition being relatively rapid (over just 100–250 years based on a sedimentation rate range of ∼10–40 cm ka^−1^ derived from our U1538‐Dove Basin stack NGR‐ties; light green sedimentation‐rate curve in Figure 8a), and thus that they represent notable iceberg rafting events. Based on our age model (Figures 7 and 8), probably no more than ∼7000 years passed between the deposition of the oldest and youngest of these three IBRD‐rich layers.

While future work should focus on establishing the provenance of all three of the discrete IBRD‐rich layers that we report from U1538, the similar sedimentological make‐up of their coarse fraction points toward a common iceberg‐rafting source. Icebergs shed from the numerous ice‐shelf fronts of the AIS today and during past glacials can contain relatively little IBRD because such material is scrubbed out of glaciers at their marine grounding lines (Alley et al., 1989). Regardless of whether the IBRD in these three discrete layers was ultimately sourced from the Weddell Sea Embayment or Amundsen Sea Embayment, we therefore propose that they may represent the first IBRD‐based evidence of a multiple stage break‐up of WAIS ice shelves (in the Amundsen Sea and/or Weddell Sea embayments) in the early Pleistocene in response to regional ocean‐atmospheric warming. Numerical modeling of the LGM highlights that Antarctic iceberg trajectories may take a more equatorward route through Iceberg Alley during cold stages following Antarctic Polar Front migration northwards (Starr et al., 2021). This observation highlights that the discrete IBRD‐rich layers that we report from our study site could owe their origin to iceberg survivability rather than increased iceberg production. We find it unlikely, though, that iceberg survivability determines the magnitude of iceberg rafting to our study site. This is because IBRD concentrations are higher overall in sediments deposited at U1538 during the (warmer) 41‐kyr (inter)glacial world than during the (colder) 100‐kyr (inter)glacial world (Figure 2a).

Our paleomagnetic‐based U1538 age model prevents us from establishing precisely the orbital‐scale relationship in time between the opal‐rich interval that contains our IBRD layers (and is indicative of a warm regional Antarctic climate) and the global signal of benthic δ^18^O in the LR04 stack. Our NGR‐based correlations between the Dove Basin stack (Reilly et al., 2021) and U1538 allow us to infer with reasonable confidence, though, that our target IBRD‐rich layers were deposited just prior to the Cobb‐Mountain subchron reversal (1.187—1.208 Ma; Channel et al., 2016), so most likely correspond to MIS 38 or the transition between MIS 38 and MIS 37 (Figure 8). Plentiful evidence exists to show that suborbital‐scale changes in climate were an inherent feature of the ∼41‐kyr (inter)glacial world (e.g., Bailey et al., 2012, 2013, 2010; Bolton et al., 2010; Hayashi et al., 2010; Hernàndez‐Almeida et al., 2012; Hodell & Channell, 2016; Raymo et al., 1998). This evidence may reflect that Last Glacial‐like DO events were a feature of this time (which would only be the case if these suborbital‐scale changes in climate were similar in magnitude to their Last Glacial counterparts). To date, though, MIS 38 remains just one of two cold stages of the ∼41‐kyr (inter)glacial world for which definitive evidence exists of this fact (at Iberian Margin IODP Site U1385, Figure 8d; Birner et al., 2016). Temperature warmed over Antarctica during Last Glacial DO‐cooling events (Brook et al., 2005; EPICA Community Members, 2006). This contrasting behavior led to the concept of a bipolar seesaw, under which suborbital‐scale changes in Atlantic Meridional Overturning Circulation strength affect the distribution of heat between the southern and northern hemispheres (Broecker, 1998; Crowley, 1992). We tentatively propose that our three early Pleistocene IBRD layers may have been connected to Last Glacial‐like bipolar seesaw oscillations that resulted in Antarctic warming events during DO‐like northern hemisphere coolings of MIS 38 (compare Figures 8d and 8e). The number of discrete IBRD‐rich layers reported here (*n* = 3) do not match the number of DO‐like events documented in the northern hemisphere at Site U1385 during MIS 38 (*n* = 8; Figure 8d). This fact may reflect that only the largest suborbital WAIS‐sourced iceberg rafting events during MIS 38 resulted in IBRD deposition overwhelming the background flux of opal sedimentation at U1538 during this cold stage. Future generation of a high resolution IBRD‐count record from U1538 across our study interval is required to determine if additional less intense suborbitally paced iceberg rafting events to Pirie Basin (which did not form discrete IBRD‐rich and diatom poor layers at U1538) occurred during this time. Regardless, the U1538 record for MIS 38 seemingly provides the first evidence that suborbital changes in climate during the 41‐kyr (inter)glacial world were a feature of both hemispheres during this time.

Iceberg Alley IBRD records of the Last Deglaciation show that Termination 1 was associated with eight major episodes of AIS instability, known as Antarctic Iceberg Discharge (AID) events (Weber et al., 2014), that each lasted from centuries to a millennium (Weber, Golledge, et al., 2021). These events are expressed in the sediment record through increased concentrations of dispersed IBRD in opal‐rich sediments (Weber et al., 2014; Weber, Golledge, et al., 2021). IBRD concentrations appear, however, to be higher in the early Pleistocene IBRD layers that are reported here for U1538. Collectively, AID events during Termination 1 appear to have been associated with Weddell Embayment grounding line retreat from its shelf edge position during the LGM to its present day location, a distance ∼800 km inland (Hillenbrand et al., 2014). If our U1538 IBRD layers are the product of the early Pleistocene equivalents to last deglacial AID events, then their relatively high IBRD concentrations may indicate they were also associated with large magnitude WAIS grounding line retreats. It is unlikely that the WAIS grounding line sat at the shelf edge in either the Weddell Sea or Amundsen Sea embayments regularly during early Pleistocene cold stages (Pollard & DeConto, 2009). The relatively high IBRD concentrations in sediments deposited at U1538 during these early Pleistocene AID‐like events are therefore more likely to reflect that the WAIS grounding line sat near to its modern‐day location during cold stages at this time and retreated further inland during these events than the position it currently occupies. Extensive ice‐shelf‐free tidewater glacier terminuses in modern‐day Alaska are associated with some of the highest marine terrigenous‐fueled sedimentation rates observed on Earth (∼40–160 m/My; Cowan et al., 2020; Gulick et al., 2015; Montelli et al., 2017). We speculate that a similar situation may have also been common for glaciers on West Antarctica during the early Pleistocene where ice‐shelf break‐up led to the existence of extensive tidewater glacier terminuses and relatively IBRD‐rich (or “dirty”) icebergs being discharged to the Southern Ocean (also see Simões Pereira et al., 2018).

Limited core recovery below the stratigraphic depths of these discrete IBRD‐rich layers prevents us from working out whether other AID‐like layers of this nature were also deposited at U1538 during older intervals of the earliest Pleistocene and the Pliocene (Figure 2). These layers are, though, found at the stratigraphic top of a ∼366‐m thick Pliocene and earliest Pleistocene sequence that appears to be much more dropstone‐ and gravel‐iceberg‐rafted debris‐rich than the upper ∼307 m of the U1538 record (Figure 2; also see Section 2); an observation we contend may be consistent with the notion that the WAIS mass‐balance was highly dynamic throughout the 41‐kyr (inter)glacial world and regularly retreated and re‐advanced inland/from its interior. This suggestion is broadly consistent with model‐ (e.g., Figure 7d; de Boer et al., 2014, 2015; Pollard & DeConto et al., 2009) and sedimentological‐ (Figure 7e; Naish et al., 2009) based evidence that the WAIS was prone to collapse during the Pliocene and earliest Pleistocene, but also with marine core‐based evidence for a highly dynamic WAIS in the Amundsen Sea Embayment region during the Pliocene (e.g., Gohl et al., 2021).

We have attributed the relatively high IBRD concentrations in early Pleistocene U1538 sediments to “dirty” icebergs calved from a West Antarctica that featured more tidewater‐based glaciers than it did during the mid‐ to late‐Pleistocene. IBRD concentrations are, however, higher in early Pleistocene U1538 sediments deposited in Pirie Basin than at U1536/7 in the more southerly Dove Basin (Figure 8b). Bulk sedimentation rates were also twice as fast at U1538 compared to U1536/7 at this time (Figure 8a), suggesting that IBRD flux to Pirie Basin was greater than it was to Dove Basin during this time. Although we cannot rule it out, as discussed above, we find it unlikely that this observation is consistent with greater AIS iceberg survivability compared to the mid‐ and late‐Pleistocene (so that Iceberg Alley icebergs melted preferentially in Pirie Basin during the ∼41‐kyr (inter)glacial world). Alternatively, IBRD deposition rates may have been relatively high at U1538 during the Pliocene and early Pleistocene because more icebergs sourced from the Pacific‐facing sector of the WAIS were delivered to Pirie Basin through Drake Passage at this time. If tidewater glacier terminuses did exist on West Antarctica during the 41‐kyr (inter)glacial world they may have generated a greater proportion of icebergs smaller than the largest tabular‐type icebergs typically calved today from its ice‐shelf fronts. This possibility is important to highlight because Antarctic iceberg modeling shows that smaller icebergs calved from, for example, the Amundsen Sea Embayment may be more likely to transit in a clockwise direction to Iceberg Alley through Drake Passage than in an anti‐clockwise direction in the Antarctic Surface Coast Current (Rackow et al., 2017). Future high‐resolution spatial‐reconstructions of IBRD deposition and surface current migrations in the Scotia Sea over the past ∼3.3 Myr are required to test between these possibilities.

## 6 Conclusions

Plio‐Pleistocene sediments recovered during IODP Expedition 382 in 2019 from Pirie Basin in the Scotia Sea provide an opportunity to gain important new insights into West Antarctic Ice Sheet evolution during the Quaternary. To this end, we present a microCT‐ and scanning electron microscope‐based sedimentological analysis and ^40^Ar/^39^Ar‐based provenance study of what is to our knowledge the first discrete IBRD‐rich sedimentary layers to be reported from early Pleistocene‐aged Iceberg Alley sediments.

This layer is the oldest of three discrete centimeter‐scale layers preserved in otherwise diatom‐opal‐rich sediments from IODP Site U1538, which we infer were deposited rapidly at ∼1.2 Ma and probably during marine isotope stage (MIS) 38 or the MIS 38‐37 transition during a warm regional climate. We further infer that it represents the sedimentary product of an intense but short‐lived episode of AIS‐iceberg rafting and IBRD deposition. Based on its sand fraction petrography and the Phanerozoic hornblendes and mica it contains, we conclude that these IBRD layers were sourced from the Atlantic‐facing Weddell Sea Embayment and/or the Pacific‐facing Amundsen Sea Embayment region of West Antarctica. They may thus provide the first IBRD‐based evidence of an early Pleistocene multiple stage break‐up of ice shelves in these regions in response to regional ocean‐atmospheric warming. We attribute their relatively high IBRD concentrations to “dirty” icebergs calved from the WAIS following retreat inland of the current location of its grounding line.

Incomplete core recovery below the stratigraphic depths of these layers prevents us from determining whether other layers like these were deposited at U1538 during older stages of the Pliocene and earliest Pleistocene, a time interval for which regular WAIS collapses are inferred. These layers are nevertheless found at the stratigraphic top of a ∼366‐m thick Pliocene and earliest Pleistocene sequence that is much more dropstone‐ and gravel‐IBRD‐rich than the upper ∼307 m of the U1538 record; an observation we contend may be consistent with the notion that a highly dynamic WAIS was a regular feature of the 41‐kyr (inter)glacial world.

## Supporting information

Supporting Information S1

Movie S1

## Data Availability

All ^40^Ar/^39^Ar ages, QEMSCAN SEM data, and depth‐age ties between NGR data from Hole U1538A (Bailey et al., 2022), the Dove Basin stack (Reilly et al., 2020) and all other shipboard expedition data presented here (Reilly et al., 2021) are archived in the IODP community at zenodo.org.
