# Supplementary material for: Episodes of Early Pleistocene West Antarctic Ice Sheet Retreat Recorded by Iceberg Alley Sediments
Source: Paleoceanogr Paleoclimatol. 2022 Jul 12;37(7):e2022PA004433. doi: 10.1029/2022PA004433 (PMC9544630; doi:10.1029/2022PA004433)
Supplement: Supplementary file 1 — Supporting Information S1 [file PALO-37-0-s002.pdf]

Paleoceanography and Paleoclimatology

Supporting Information for

Episodes of early Pleistocene West Antarctic Ice Sheet retreat recorded by Iceberg Alley sediments

Ian Bailey[1]*, Sidney Hemming[2], Brendan T. Reilly[3], Gavyn Rollinson[1], Trevor Williams[4], Michael E. Weber[5], Maureen E. Raymo[2], Victoria L. Peck[6], Thomas A. Ronge[7], Stefanie Brachfeld[8], Suzanne O'Connell[9], Lisa Tauxe[3], Jonathan P. Warnock[10], Linda Armbrecht[11], Fabricio G. Cardillo[12], Zhiheng Du[13], Gerson Fauth[14], Marga Garcia[15,16], Anna Glueder[17], Michelle Guitard[18], Marcus Gutjahr[19], Ivan Hernández-Almeida[20], Frida S. Hoem[21], Ji-Hwan Hwang[22], Mutsumi Iizuka[23], Yuji Kato[24], Bridget Kenlee[25], Yasmina M. Martos[26,27], Lara F. Pérez[6,28], Osamu Seki[29], Shubham Tripathi[30], Xufeng Zheng[31].

[1]Camborne School of Mines, University of Exeter, Penryn Campus, Treliever Road, Cornwall TR10 9FE, UK.
[2]Lamont-Doherty Earth Observatory, Columbia University, Palisades, NY 10964, USA.
[3]Scripps Institution of Oceanography, University of California San Diego, La Jolla, CA 92093, USA.
[4]International Ocean Discovery Program, Texas AM University, College Station, TX 77845, USA.
[5]Institute for Geosciences, Department of Geochemistry and Petrology, University of Bonn, Germany.
[6]British Antarctic Survey, Cambridge CB3 0ET, UK.
[7]Alfred-Wegener-Institut Helmholtz-Zentrum für Polar-und Meeresforschung.
[8]Earth and Environmental Studies, Montclair State University, Montclair, NJ 07043, USA.
[9]Department of Earth and Environmental Sciences, Wesleyan University, Middletown, CT 06459, USA.
[10]Department of Geoscience, Indiana University of Pennsylvania, Indiana, PA 15705, USA.
[11]Institute for Marine and Antarctic Studies, University of Tasmania, Battery Point TAS 7004, Australia
[12]Departmento Oceanografia, Servicio de Hidrografia Naval, Ministerio de Defensa, Argentina.
[13]State Key Laboratory of Cryospheric Science, Northwest Institute of Eco-Environment and Resources, Lanzhou 730000, China.
[14]Geology Program, University of Vale do Rio dos Sinos, San Leopoldo RS 93022-750, Brazil.
[15]Andalusian Institute of Earth Science (CSIC-UGR). Armilla (Granada) 18100, Spain.
[16]Cádiz Oceanographic Centre (IEO-CSIC), Cádiz 11006, Spain.
[17]College of Earth, Ocean, and Atmospheric Sciences, Oregon State University, Corvallis, OR 97331, USA.
[18]College of Marine Science, University of South Florida, St. Petersburg, FL 33701, USA.
[19]GEOMAR, Helmholtz Centre for Ocean Research, University of Kiel, 24148 Kiel, Germany.
[20]Department of Earth Sciences, ETH Zurich, Sonneggstrasse 5, 8092 Zurich, Switzerland.
[21]Department of Earth Science, Marine Palynology and Paleoceanography, Utrecht University, 3584 CB Utrecht, Netherlands.
[22]Earth Environmental Sciences, Korea Basic Science Institute, Chungbuk Cheongju, Republic of Korea.
[23]Knowledge Engineering, Tokyo City University, Tokyo setagaya-ku 158-0087, Japan.
[24]Faculty of Life and Environmental Sciences, University of Tsukuba, Tsukuba, Ibaraki 305-8572.
[25]Department of Earth Sciences, University of California Riverside, Riverside, CA 92521, USA.
[26]NASA Goddard Space Flight Center, Planetary Magnetospheres Laboratory, Greenbelt, MD 20771, USA.
[27]University of Maryland, Department of Astronomy, College Park, MD 20742, College Park, USA.
[28]Geological Survey of Denmark and Greenland, Department of Marine Geology, Aarhus University City 81, building 1872, 6 floor, DK-8000, Denmark.
[29]Institute of Low Temperature Science, Hokkaido University, Sapporo Hokkaido 060-0819, Japan.
[30]Marine Stable Isotope Lab, National Centre for Polar and Ocean Research, Ministry of Earth Sciences, Vasco. Da Gama 403804, India.
[31]South China Sea Institute of Oceanology, Chinese Academy of Sciences, Guangzhou 510301, China.

Contents of this file

Figures S1 to S5

Additional Supporting Information (Files uploaded separately)

Caption for Table S1
Captions for Data Sets S1-S5 (Bailey et al., 2022)
Caption for Movie S1 (Bailey et al., 2022)

Introduction

This supporting information document contains five figures referred to in the main text. It also contains Table S1, and caption explainers of Data Sets S1–S5 (of our $^{40}Ar/^{39}Ar$ age and QEMSCAN SEM data and U1538-Dove Basin Stack NGR ties) and the Movie S1 that are archived in the IODP community at zenodo.org (Bailey et al., 2022):

https://zenodo.org/record/6628734#.YqIY8GApA80.

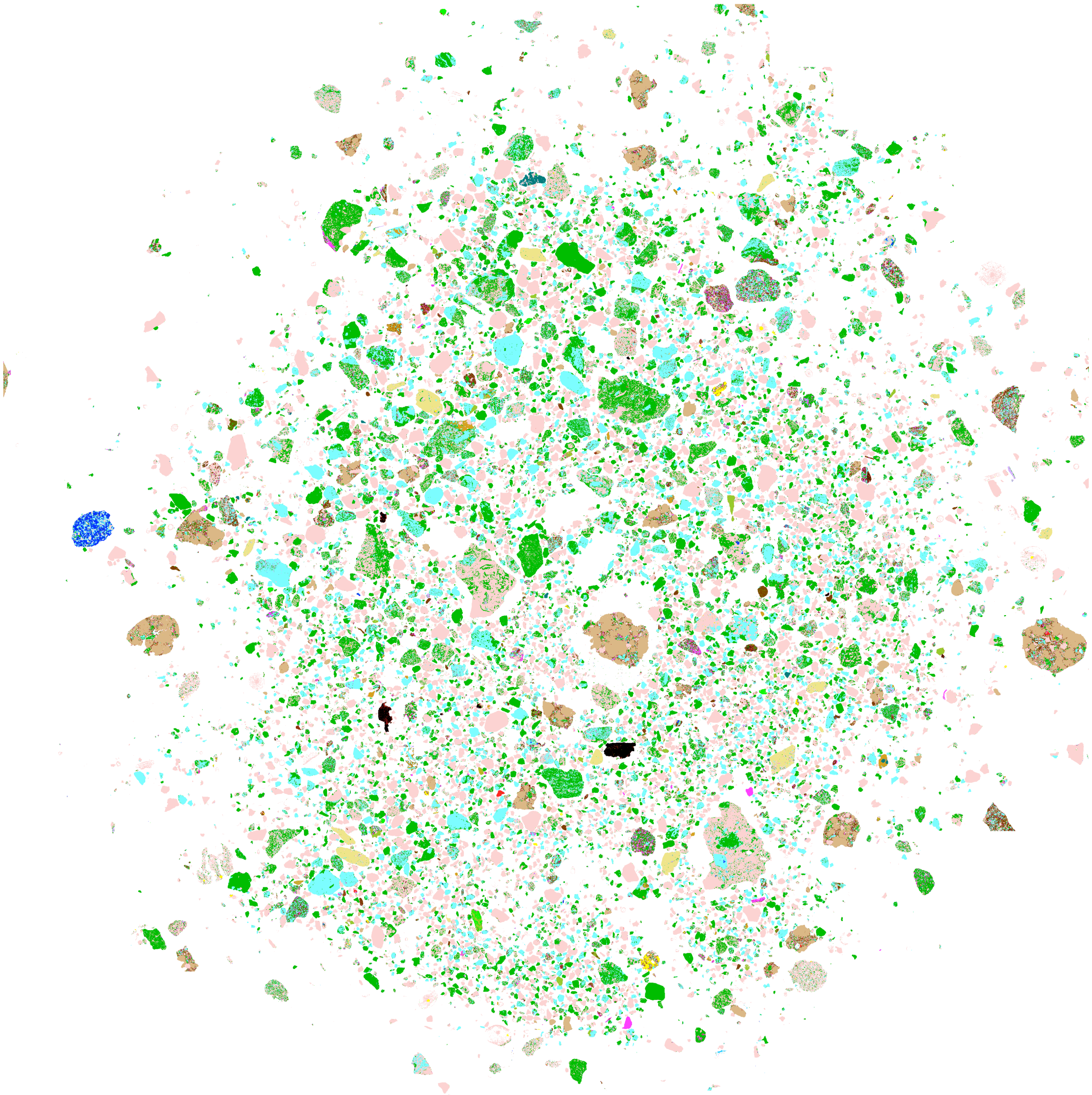

Figure S1. QEMSCAN-derived mineral map of sand-sized fraction of Hole U1538A 36X-3W-47–49 cm. Image is 30-mm wide. See key in Data Set S1 for color guide to the sample mineralogical make-up shown.

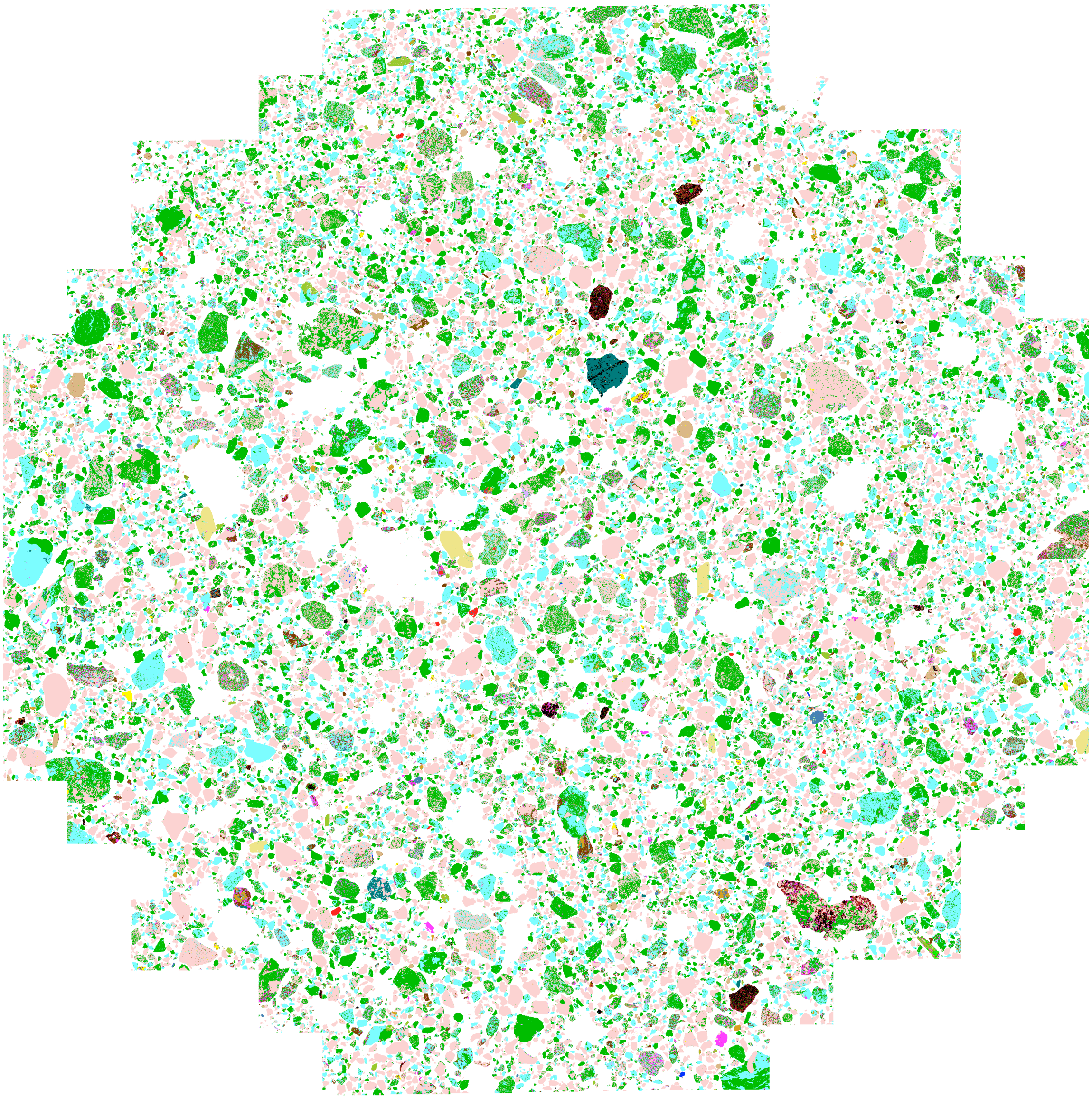

Figure S2. QEMSCAN-derived mineral map of sand-sized fraction of Hole U1538A 36X-3W-49–50 cm. Image is 30-mm wide. See key in Data Set S1 for color guide to the sample mineralogical make-up shown.

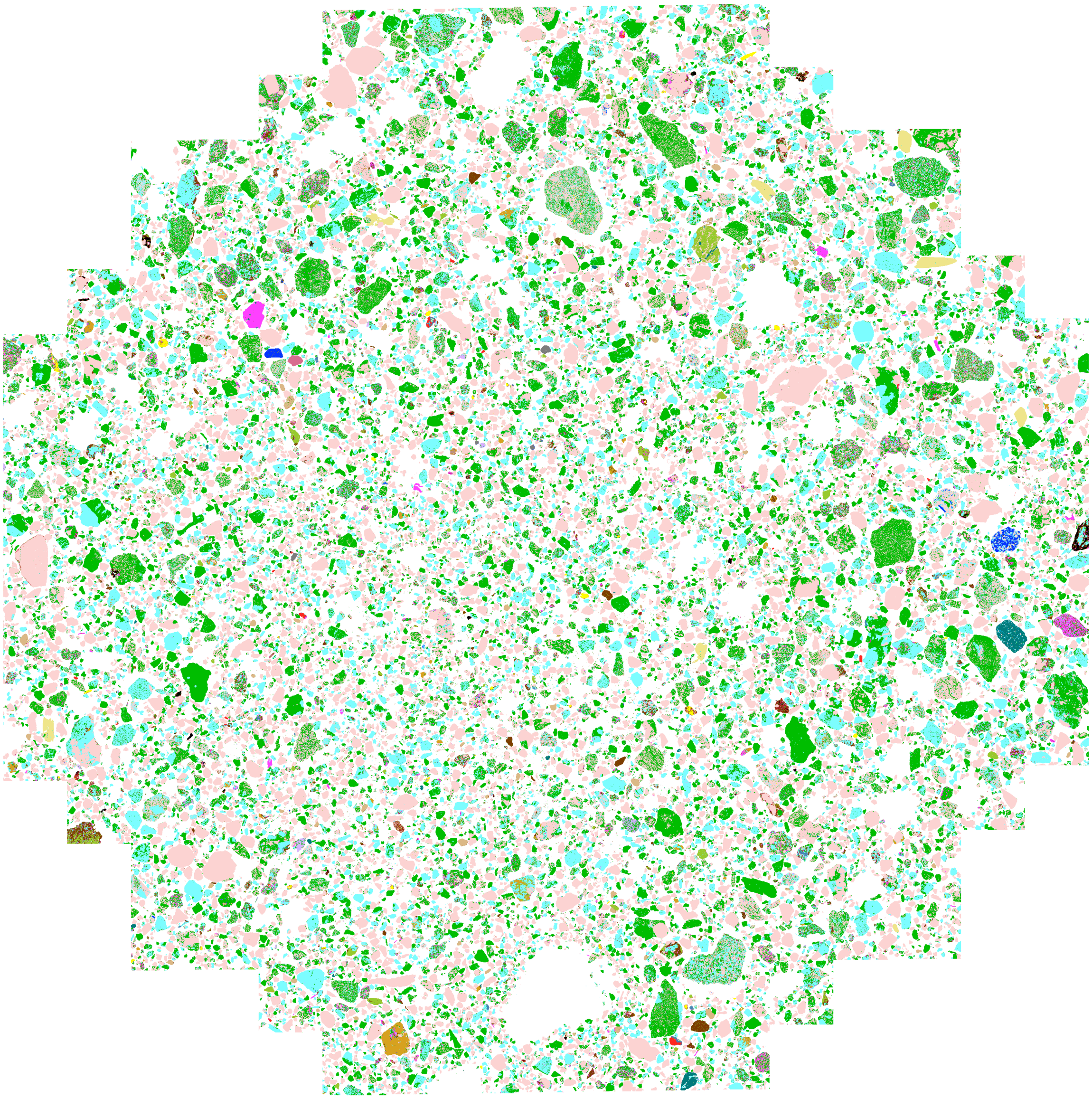

Figure S3. QEMSCAN-derived mineral map of sand-sized fraction of Hole U1538A 36X-3W-50–51 cm. Image is 30-mm wide. See key in Data Set S1for color guide to the sample mineralogical make-up shown.

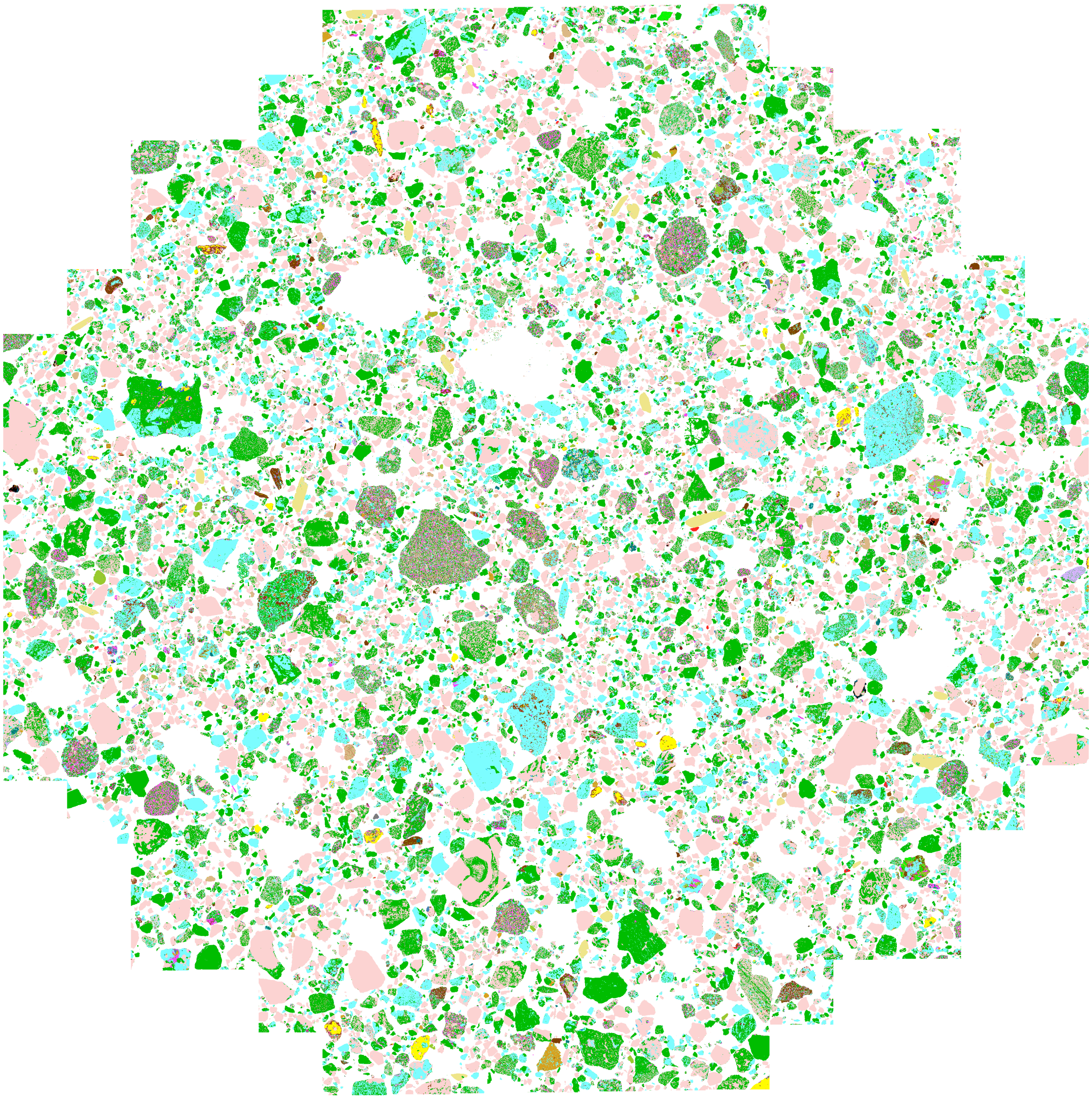

Figure S4. QEMSCAN-derived mineral map of sand-sized fraction of Hole U1538A 36X-3W-51–52 cm. Image is 30-mm wide. See key in Data Set S1 for color guide to the sample mineralogical make-up shown.

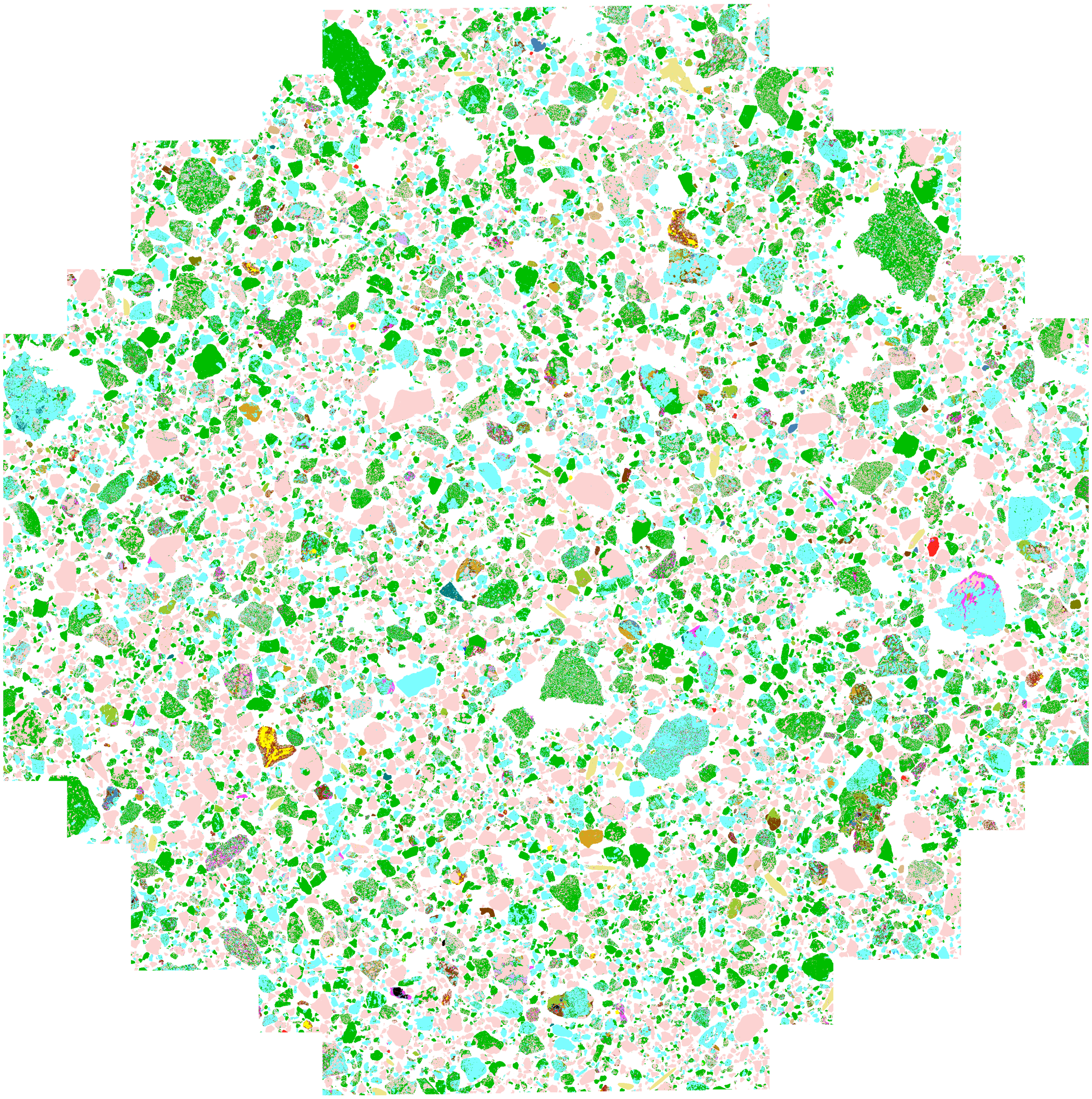

Figure S5. QEMSCAN-derived mineral map of s sand-sized fraction of Hole U1538A 36X-3W-52–53 cm. Image is 30-mm wide. See key in Data Set S1 for color guide to the sample mineralogical make-up shown.

Table S1. Mineral List – a guide to mineral names stated in Data Sets S1 and S2 and their QEMSCAN® chemistry-based descriptions.

Mineral category	QEMSCAN chemistry-based mineral description
Background	All mounting media related edge effects
Fe sulphides	Includes Pyrite, Pyrrhotite, and Fe sulphates (weathered sulphides)
Rutile	Any phase with Ti,O (Rutile/Anatase/Brookite).
Ilmenite	Any phase with Fe,Ti,O.
Titanite	Any phase with Ca,Ti,Si,O and minor Al,F,Fe.
Mn Garnet	Any phase with Mn,Fe,Al,Si,O (Spessartine)
Fe Ox/CO3	Fe oxides/carbonates such as Siderite, Hematite, Magnetite, Goethite, Limonites
Monazite	Includes Monazite (Ce,P,O other REE) & Xenotime
Zircon	Any phase with Zr,Si,O
Quartz	Quartz and other silica minerals
Plagioclase	Plagioclase Feldspars: phases with Na,Al,Si,O to Ca,Al,Si,O.
K-Feldspar	K-Feldspars (Orthoclase, Sanidine, Microcline): any phase with K,Al,Si,O.
Muscovite/Illite	Muscovite/Lepidolite Mica/Illite - any phase with K,Al,Si,O.
Biotite	Biotite Mica (Fe,Al,K,Mg,Si,O), may include other micas and clays (Fe Illite)
Glauconite	Any phase with Fe,Al,K,Mg,Si,O. Based on previous jobs with confirmed Glauconite
Tourmaline	Any phase with Fe,Al,Si,Mg,O (B not detectable by EDS).
Kaolinite	Any phase with Al,Si,O such as Kaolinite/Halloysite/Dickite
Al silicates	Any phase with Al,Si,O: Andalusite/Sillimanite/Kyanite, or Topaz with F<3%
Fe silicates (Fe-olivines)	Any phase with Fe,Si,O. May contain trace Ca.
Olivine/OPX	Any phase with Mg,Fe,Si,O. May include the Serpentine Group, Pyroxenes, Olivine
Ca Fe Al silicates (garnet/epidote)	Any phase with Ca,Fe,Al,Si,O with or without Mg. May include Epidote, Garnet
Ca Fe Mg silicates (hornblende/cpx)	Any phase with Ca,Mg,Fe,Si, (with or without Al) such as Hornblende, Diopside
Chlorite/Almandine	Any phase with Fe,Al,Si, and Fe,Al,Si Mg,O such as Chlorite and Almandine Garnet
Calcite	Any phase with Ca,C,O. Incudes minor/trace Ankerite, Dolomite
Apatite	Any phase with Ca,P,O.
Gypsum	Any phase with Ca,S,O
Barite	Any phase with Ba,S,O
Others	Any other mineral not included above. Trace Cassiterite, Chalcopyrite

Data Sets S1-S5 and Move S1 captions from Bailey et al. (2022)

Data Set S1. Modal mineralogy data based on QEMSCAN® analyses, which infer minerals from chemistry. The mineral name assignations for each chemistry-based category stated in this table are aided by visual (microscope-based) inspection of the raw sieved samples.

Data Set S2. Mineral association data based on QEMSCAN® analyses. Please read data in columns, mineral against mineral (down then across left). These data define what touches what in the sample and is displayed as a percentage. Association refers to adjacency. Two minerals are "associated" if a pixel of one of the minerals occurs adjacent to a pixel of the other mineral. iExplorer software used scans the measured particles horizontally, from left to right, counting the associations that occur in the images (so the more pixels/closer the x-ray spacing the more accurate the data). Each column is independent. That is, it is split into a percentage of what touches what, so it is not expected that any two minerals' data are reciprocal. The background category primarily reflects the free boundaries of 'grains' rather than liberated grains/particles. While it may provide an indicator of liberation, it does not represent liberation since it does not describe 'particles' which are made up of mineral grains. Inclusions and composite particles are therefore not described. Please consider the modal mineralogy (Tab. S1) when examining these mineral association data.

Data Set S3. Lithotyping data based on QEMSCAN® analyses. Particles have been digitally filtered using a set of lithotype rules (also displayed in this data set). These rules are based on the mineral grains in the particles themselves and use their area percent within each particle and their size in microns. The lithotype names stated here are largely assigned based on the dominant mineral grain in each category.

Data Set S4. $^{40}Ar/^{39}Ar$ ages of individual sand-sized hornblende and mica. See main text for method used to generate these ages.

Data Set S5. Ties to place Hole U1538A NGR data on Dove Basin Stack (Reilly et al., 2021) depths.

Movie S1. 3D-volume realization based on non-destructive X-ray microtomography imaging of a centimeter-scale iceberg-rafted debris-rich layer in Hole U1538A-36X-3W. 3D images were generated using a helical scanning trajectory that allows for long scan sequences and fast acquisition time. Based on the sample geometry, a voxel (pixel) resolution of ~14-μm was achieved. The 7000+ projection images were reconstructed to produce a 3D volume of image intensities (where higher values indicate greater x-ray attenuation). Avizo software was used for 3D segmentation and volume rendering to visualize gravel and sand to create this animation. The different colors assigned to each clast were chosen arbitrary.
